# Demographic and Structural Variability Modulate Growth Dynamics in European Beech Primary Forests

**DOI:** 10.1111/gcb.70902

**Published:** 2026-05-06

**Authors:** Krešimir Begović, Jakob Pavlin, Thomas Langbehn, Kristyna Svobodová Langbehn, Jakub Kašpar, Andrei Popa, Thomas A. Nagel, Jeňýk Hofmeister, Pavel Janda, Miloš Rydval, Daniel Kozak, Martin Mikoláš, Stjepan Mikac, Miroslav Svoboda

**Affiliations:** ^1^ Faculty of Forestry and Wood Sciences Czech University of Life Sciences Prague Prague Czech Republic; ^2^ Faculty of Environmental Sciences Czech University of Life Sciences Prague Prague Czech Republic; ^3^ Department of Forest Ecology Landscape Research Institute Brno Czech Republic; ^4^ National Institute for Research and Development in Forestry “Marin Drăcea” Bucharest Romania; ^5^ Faculty of Silviculture and Forest Engineering Transilvania University of Brasov Brasov Romania; ^6^ Biotechnical Faculty University of Ljubljana Ljubljana Slovenia; ^7^ Department of Forest Ecology and Silviculture, Faculty of Forestry University of Zagreb Zagreb Croatia

**Keywords:** basal area increment, climate response analysis, dendroecology, drought legacies, *Fagus sylvatica*, generalized mixed‐effect model, growth trends, superposed epoch analysis

## Abstract

Intensifying droughts and heatwaves are increasing hydraulic constraints on European beech ( *Fagus sylvatica* L.) and driving recent tree vitality losses at its warmer and drier range margins. Network‐wide growth models predict widespread beech growth declines under future warming, yet these models rarely account for intraspecific demographic variability, potentially underestimating the adaptive capacity of natural beech forests. We leveraged a network of 530 plots and ~11,000 trees from primary beech forests covering a broad environmental gradient in central and southeastern Europe to assess how demographic variability in growth dynamics modulates forest productivity. We explored demographic differences in temporal and spatial variation in radial growth patterns and growth sensitivity to climate warming. Previous summer maximum temperatures and climatic water balance were the dominant climatic constraints of radial growth in xeric regions, whereas growth in mesic regions was more closely associated with current spring and summer conditions. Large trees exhibited stronger sensitivity to prior‐summer heat stress, while small trees had stronger associations with spring and summer moisture availability. Severe summer droughts caused pronounced multi‐year growth legacies across the demographic strata only in xeric regions. Long‐term growth trend analysis revealed substantial regional heterogeneity, with growth declines in mid‐sized and large trees contrasted by gains in small and mid‐sized young (mesic regions) and old trees (xeric regions). Recent growth declines were linked to rising summer temperatures and declining water balance across the forest strata. Overall, productivity losses outweighed gains across the study network under climate warming. Stand structural complexity and demographic variability in growth dynamics were key drivers of ecosystem productivity, but their positive effects diminished under severe climatic stress. Our findings highlight the critical role of demographic and structural heterogeneity in shaping beech growth dynamics under climate warming, and caution against inferring future ecosystem productivity trajectories without explicitly accounting for demographic variability.

## Graphical abstract

Climate change is increasing drought and heat stress on European beech ( *Fagus sylvatica* L.), causing widespread tree vitality losses and growth declines across Europe. Using a large tree ring network from montane primary beech forests, we found that trees of various ages and sizes respond differently to climate warming, leading to contrasting within‐population growth trends along environmental gradients. Structurally complex forests better maintain productivity under shifting climatic constraints, but this buffering effect diminishes under extreme climatic stress, particularly in drier regions. Our findings collectively caution against inferring future ecosystem productivity trajectories under climate change without explicitly accounting for demographic variability.

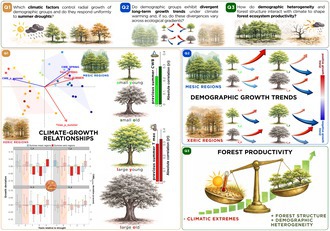

## 1 Introduction

Global climate change has been altering fundamental processes that regulate natural disturbance regimes and forest dynamics, including tree growth, recruitment and mortality rates (Altman et al. 2024; Cerioni et al. 2024; Martini et al. 2024). Droughts, heatwaves, and other extreme climatic events have become the main culprits of widespread tree vitality losses reflected by growth declines and elevated mortality rates across temperate forest ecosystems (Allen et al. 2010, 2015; Anderegg et al. 2015; Schuldt et al. 2020; Senf et al. 2018, 2020; Spinoni et al. 2015). The predicted rise in frequency and duration of compound climatic extremes will likely exacerbate climate‐driven mortality risks in the future (Bastos et al. 2021; IPCC 2023) and negatively affect the long‐term forest ecosystem functioning and stability, with cascading effects on forest productivity and ecosystem services (Adams et al. 2017; Anderegg et al. 2020; Kannenberg et al. 2022). Understanding the impacts of changing environmental conditions on contemporary forest dynamics across biogeographic regions is, therefore, a critical research priority in global climate change studies.

Montane beech‐dominated forests are among temperate forest types expected to undergo major structural and functional changes in the coming decades (Bussotti et al. 2015; Hacket‐Pain et al. 2016). European beech ( *Fagus sylvatica* L.) is one of the most economically and ecologically important tree species in Europe (Geßler et al. 2006), both as a major timber source (Duncker et al. 2012; Pramreiter and Grabner 2023) and in forest conversion strategies (e.g., Löf et al. 2023). Natural beech forests provide multiple essential ecosystem services, including biodiversity conservation (Kameniar et al. 2023; Kozák et al. 2023; Mikoláš et al. 2021) and large carbon storage (Keith et al. 2024; Ralhan et al. 2023). The species' broad latitudinal and elevational distribution across the European temperate zone reflects its high acclimation capacity in response to environmental variability (Leuschner and Ellenberg 2017). However, recent canopy diebacks and growth declines observed at the species' warmer and drier range margins (e.g., Arend et al. 2022; Buras et al. 2020; Gribbe et al. 2024; Klesse et al. 2022) raise concerns regarding beech ecosystem resilience and carbon sink role under climate change.

Growth modelling efforts from extensive tree‐ring networks suggest that beech productivity will decline across most of its distribution range under warmer climate change scenarios (Dorado‐Liñán et al. 2022; Klesse et al. 2024; Martinez del Castillo et al. 2022), particularly in drier regions where hydraulic constraints exceed the species' physiological thresholds (Jump et al. 2006; Peñuelas et al. 2008). In contrast, growth increases are expected mainly in wetter and colder montane regions, where rising spring temperatures may alleviate cold limitations and extend the growing season (Bošela et al. 2023; Kasper et al. 2023; Serra‐Maluquer et al. 2018). These climate‐driven growth projections are valuable tools for making robust inferences about future forest productivity trajectories under climate change, but most large‐scale growth models predominantly rely on large canopy‐dominant trees for inferring forest ecosystem responses to environmental variation. Large trees have a disproportionately higher contribution to forest biomass and ecosystem functioning (Mildrexler et al. 2020; Stephenson et al. 2014), and are therefore commonly treated as representative of stand‐level growth dynamics. However, focusing solely on canopy‐dominant trees ignores the potential contribution of suppressed and intermediate‐sized trees to shaping forest structure and productivity under rising climate stress. Moreover, this common approach implicitly assumes demographic homogeneity in tree growth sensitivity to climatic variation, which may fail to fully capture climate change impacts on forest ecosystem functioning and, ultimately, limit robust estimates of future forest productivity (Babst et al. 2018; Peltier and Ogle 2020). Quantifying within‐population growth patterns could help refine cross‐regional growth models and improve predictions of beech ecosystem responses to climate change, but the required species‐specific empirical analyses at broad ecological scales remain rare.

Climate sensitivity of a tree species varies not only spatially across local environmental conditions, but also within a forest population across the demographic strata. Functional traits associated with variations in tree age, size, and life histories underpin the potential of individual trees for an adaptive response to environmental variability (Anderson‐Teixeira et al. 2022; Violle et al. 2012; Zang et al. 2014), which, in turn, shapes the adaptive capacity of the entire forest population. For example, large trees are commonly more vulnerable to short‐ and long‐term changes in climatic conditions due to greater hydraulic demands and mechanical constraints under heightened evaporative pressures (Bennett et al. 2015; McDowell and Allen 2015), whereas smaller trees, although more competitively suppressed, are climatically buffered in the subcanopy and often exhibit highly plastic responses to stochastic environmental variation with divergent growth patterns (Hertel et al. 2013; Rollinson et al. 2021). Large trees typically exhibit a stronger common environmental signal (Alexander et al. 2018; Au et al. 2022), but the assumption that their uniform response to climatic variability accurately represents forest ecosystem dynamics can be misleading (Bowman et al. 2013; Clark et al. 2011; Nehrbass‐Ahles et al. 2014).

Adopting a stratified approach when assessing forest ecosystem responses to climate change may mitigate some of the shortcomings of large‐scale models, particularly in environmentally heterogeneous areas where model accuracy declines (e.g., Klesse et al. 2024), and help refine ecological interpretations of ecosystem resilience under climate warming (Matsushita et al. 2015; Scholes 2017; Voelker 2011). This can be particularly useful in studies of unmanaged forests where long‐term fine‐scale environmental variation and disturbance processes introduce large demographic variability into the forest structure. As a result, these forests are often characterized by heterogeneous age and size structures with multi‐layered canopies (Meigs et al. 2017; Nagel et al. 2014), which collectively facilitate stable long‐term carbon storage and higher ecosystem resilience to external perturbations compared to managed forests (Keith et al. 2009; Luyssaert et al. 2008). To our knowledge, no study has yet systematically assessed how intraspecific demographic variation in climate sensitivity and forest structure affects beech ecosystem productivity under climate change across biogeographic regions. Recent cross‐regional dendroecological studies show that beech radial growth sensitivity to rising aridity and climate warming is spatially and temporally variable across broad environmental gradients (Kašpar et al. 2024; Leifsson et al. 2024; Serrano‐Notivoli et al. 2025; Tumajer et al. 2023), but it remains unclear to what extent demographic variability modulates these spatiotemporal divergences and how it influences beech ecosystem productivity under climate change.

Driven by these knowledge gaps and contemporary trends observed across temperate beech forests over recent decades, we aimed to explore how beech growth dynamics vary among different levels of demographic strata under climate warming, and whether demographic heterogeneity alters forest productivity estimates along environmental gradients. To that end, we leveraged an extensive tree‐ring dataset from primary beech forests in central and southeastern Europe. Pan‐regional tree‐ring networks span broad ecological gradients and facilitate robust analyses of growth responses to climatic variation at various spatiotemporal scales (Dobbertin 2005; Fritts 1976). Although relatively scarce due to extensive historical land use and forest management practices (Sabatini et al. 2018, 2020), primary forests are an ideal natural reference for quantifying demographic growth responses to climate change, as their demographic compositions are shaped in the absence of direct anthropogenic impacts.

Our overarching aim is to contribute to the growing body of literature on beech ecosystem resilience to climate change by explicitly testing whether demographic heterogeneity alters generalizations about climate‐growth relationships, long‐term growth trajectories, and forest productivity trends across beech primary forests under a warmer and drier climate. To achieve this, trees were a priori categorized into predetermined demographic groups based on their age and size (DBH—diameter at breast height) at the time of sampling in order to capture a spatiotemporal snapshot of stratified growth patterns across biogeographic regions. Age and size distributions varied between regions, but encompassed the full demographic spectrum of beech forest populations (Figures S1 and S2). We addressed the following research questions:

Which climatic factors most strongly control radial growth of different demographic groups under recent climate warming, and do demographic groups respond uniformly to severe summer droughts across ecological gradients?

Central and southeastern Europe have experienced substantial warming and rising frequency of severe droughts since the 1980s (Naumann et al. 2018). If age‐ and size‐specific demographic groups diverge in their growth responses to multi‐decadal warming and stochastic climatic anomalies, model predictions relying primarily on large trees may mischaracterize both short‐term tree growth responses and long‐term forest productivity trends.

Do demographic groups exhibit divergent long‐term growth trends under recent climate change and, if so, do these divergences vary across ecological gradients?

If demographic groups systematically differ in their growth response to climate warming and extreme droughts, then regional growth trajectories may also be demographically structured. Evaluating whether interactions between ontogenetic traits and shifting climatic constraints lead to divergent within‐population growth trends provides an empirical assessment of the predicted climate‐driven growth declines across beech forests under climate change.

How does demographic variability in growth dynamics and forest structure interact with climate variability to shape forest ecosystem productivity under climate warming?

Disentangling the relative contributions of demographic growth dynamics, forest structure, and climatic variation to ecosystem productivity can reveal whether demographic heterogeneity mitigates climate‐driven beech declines and alter projections of future beech forest trajectories, which would have profound implications for future carbon sink role of beech ecosystems.

## 2 Materials and Methods

### 2.1 Study Area and Climate Data

**FIGURE 1:**
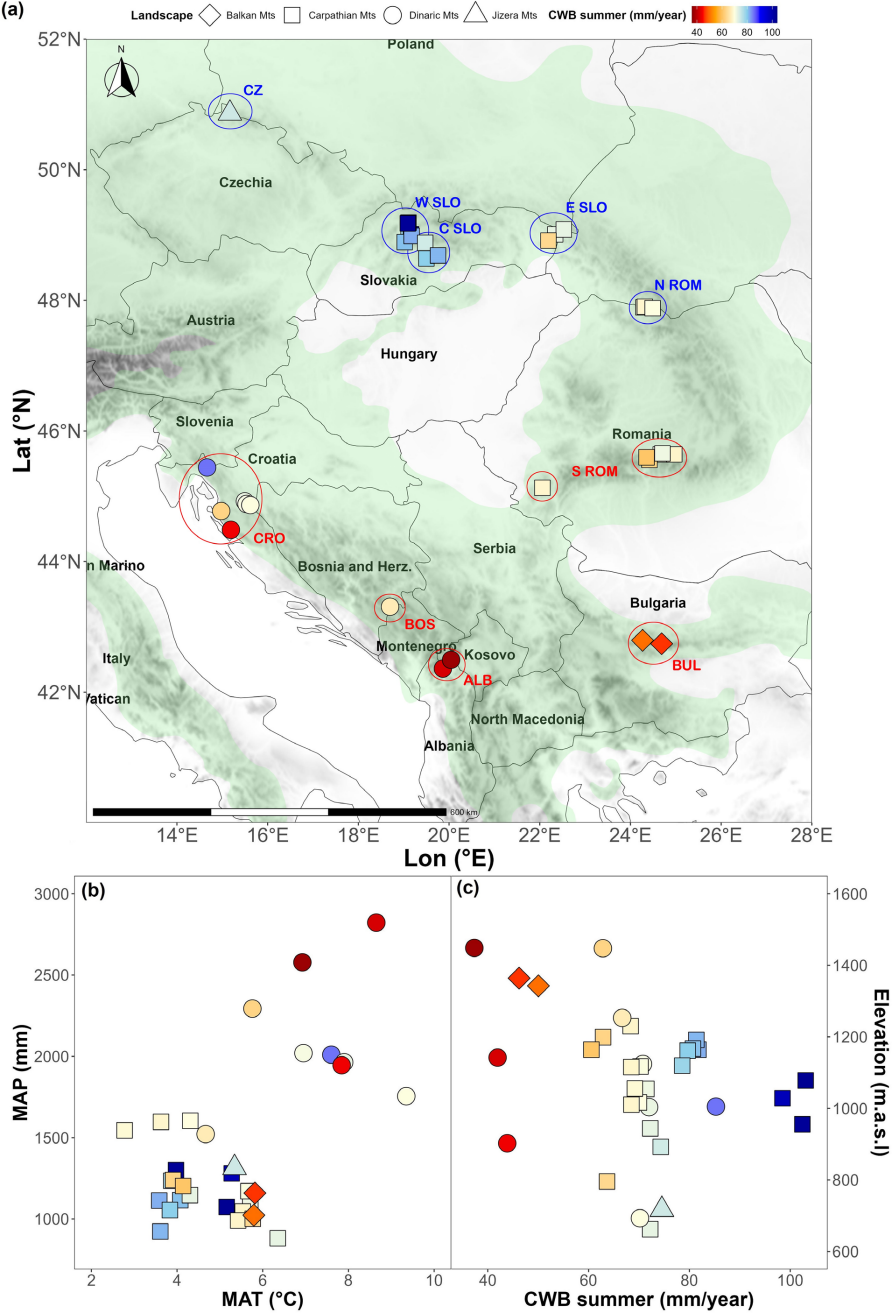
Spatial and bioclimatic gradients of the study area. (a) Geographical distribution of study sites within the natural distribution range of European beech (Caudullo et al. 2017; green area), distributed along the climatic (b) and elevational (c) gradient. Mean annual temperature (MAT) and precipitation totals (MAP) were obtained for the coordinates of individual sites over the period of accelerated climate warming (1980–2020). Colors depict differences in mean annual summer (i.e., June–August) climatic water balance (CWB = Precipitation—Potential Evapotranspiration), with warmer colors (yellow‐to‐red) showing drier sites and cooler colors (light‐to‐dark blue) wetter sites, whereas shapes denote individual biogeographic regions, respectively. Regional acronyms denote regions as defined in Table 1. Map lines delineate study areas and do not necessarily depict accepted national boundaries.

The study was conducted along a broad latitudinal (42.8°–50.9° N) and elevational gradient (615–1688 m a.s.l.) of European beech primary forests, covering a substantial portion of the species' natural distributional range in central and southeastern Europe (Figure 1). The study area is part of an international network of permanent forest inventory plots established in the dominant mountain ranges of central and southeastern Europe (i.e., REsearch on MOuntain TEmperate network; see www.remoteforests.org), and includes regions in the Carpathian Mts. (Slovakia/SLO, Romania/ROM), Dinaric Mts. (Croatia/CRO, Bosnia/BOS, Albania/ALB), Balkan Mts. (Bulgaria/BUL) and the Jizera Mts. (Czechia/CZ). The study area represents beech‐dominated mixed forest stands with a significant share (> 10% basal area) of silver fir ( *Abies alba* Mill.) and sycamore maple ( *Acer pseudoplatanus* L.), and minor admixing of Norway spruce ( *Picea abies* L.), Norway maple ( *Acer platanoides* L.), European ash ( *Fraxinus excelsior* L.), small‐leaved linden ( *Tilia cordata* Mill.), common hornbeam ( *Carpinus betulus* L.), Bosnian maple (in the southern regions; *Acer obtusatum* Waldst. et Kit. ex Willd.), and Wych elm (in the southeastern regions; *Ulmus glabra* Huds.). Forest stands were assessed for signs of naturalness (e.g., presence of large old trees, diverse horizontal, vertical, and age structures, significant amounts of standing and downed deadwood) during field reconnaissance, and validated with available historical information from local sources (e.g., expert local knowledge, aerial photographs) prior to establishing individual sampling plots.

Forest stands were broadly distributed within the species' climatic envelope, with mean annual precipitation ranging from 881 to 2822 mm and mean annual temperature from 2.8°C to 9.4°C (Figure 1, Table 1). Climate data were obtained from the high‐resolution (1 km^2^) CHELSA CRU timeseries dataset (Karger et al. 2018), which combines interpolation and statistical downscaling approaches with orographic predictors to improve computation of temperature and precipitation estimates. Climate data were extracted for the coordinates of each forest stand over the period 1940–2020, covering time intervals before and after the onset of accelerated climate warming observed across the network (Figure S3a). We extracted monthly minimum (T_min_) and maximum temperature (T_max_), as well as precipitation sums (Prec), with which we calculated climatic water balance (CWB) using the modified Hargreaves' formula (Droogers and Allen 2002; Hargreaves 1994) to capture site‐specific soil moisture conditions. Stands were then categorized according to summer climate conditions under climate warming as “summer‐mesic” or “summer‐xeric” based on the principal component analysis (PCA; Jolliffe 2002) and loading of the first principal component (Figure S3b).

**TABLE 1:** Study area information.

Country	Code	Stand	Lat	Lon	Elevation	MAT	MAP	CWB summer	Nplot	Ntree	Age	DBH
Czech Republic	CZ	Jizera Mountains	50.86	15.17	594–840	5.34	1314.40	74.54	23	438	142 (±85)	50.7 (±32.1)
Slovakia	W SLO	Kundracka	49.01	19.16	963–1193	5.16	1073.40	103.07	7	85	242 (±105)	41.6 (±25.7)
Slovakia	W SLO	Sutovska	49.18	19.09	769–1142	3.98	1300.00	102.41	14	420	138 (±105)	31 (±30.5)
Slovakia	W SLO	Sramkova	49.19	19.10	943–1114	5.27	1280.50	98.47	14	378	122 (±76)	24.5 (±26.9)
Slovakia	W SLO	Skalna Alpa	48.99	19.17	1106–1222	3.58	1113.10	81.78	8	112	192 (±186)	37.1 (±34)
Slovakia	W SLO	Padva	48.89	19.02	1064–1270	4.08	1115.60	80.69	7	108	166 (±62)	32.2 (±15.9)
Slovakia	W SLO	Kornietova	49.00	19.11	1013–1226	3.87	1234.60	78.57	14	383	172 (±100)	27.8 (±25.6)
Slovakia	C SLO	Klenovsky Vepor	48.69	19.76	1112–1271	3.61	923.30	81.38	13	182	124 (±126)	31.4 (±33.2)
Slovakia	C SLO	Polana	48.64	19.50	1038–1285	3.83	1054.80	79.66	20	295	144 (±65)	38 (±25)
Slovakia	C SLO	Obrstin	48.88	19.47	832–953	5.62	1005.90	74.32	6	123	90 (±94)	38.6 (±34.3)
Slovakia	E SLO	Stuzica	49.09	22.53	820–1068	4.30	1146.30	72.23	42	957	175 (±124)	39.4 (±34.5)
Slovakia	E SLO	Havesova	49.01	22.34	615–710	6.35	881.60	72.22	14	151	222 (±98)	65.8 (±34.4)
Slovakia	E SLO	Vihorlat	48.91	22.19	643–948	5.77	1001.40	63.66	21	364	158 (±108)	43.3 (±43.2)
Romania	N ROM	Paulic	47.88	24.49	936–1097	4.31	1603.50	69.99	12	270	202 (±57)	45.5 (±25)
Romania	N ROM	Bistra valley	47.89	24.29	959–1152	2.78	1545.00	69.18	14	248	162 (±91)	42 (±33.4)
Romania	N ROM	Criva	47.90	24.32	874–1147	3.62	1596.70	68.49	14	249	173 (±96)	45 (±34.7)
Romania	S ROM	Ucea Mare	45.66	24.71	923–1186	5.71	1098.40	71.46	14	227	167 (±100)	49.4 (±30)
Romania	S ROM	Arpasul	45.64	24.67	1021–1213	5.67	1171.10	70.30	14	253	171 (±90)	34.7 (±32.2)
Romania	S ROM	Izvoarle Nerei	45.13	22.06	994–1237	5.53	1046.00	68.47	14	281	244 (±116)	55.7 (±36.2)
Romania	S ROM	Belia	45.64	24.97	1159–1301	5.42	990.20	68.34	14	331	116 (±110)	35.7 (±30)
Romania	S ROM	Boia Mica	45.56	24.42	1127–1271	3.91	1239.50	62.87	14	460	155 (±78)	39.4 (±39.8)
Romania	S ROM	Sebesu	45.60	24.36	1004–1324	4.14	1202.80	60.55	14	447	146 (±112)	38.6 (±26.7)
Croatia	CRO	Risnjak	45.44	14.67	931–1079	7.60	2010.00	85.27	14	114	270 (±117)	52 (±25.5)
Croatia	CRO	Corkovo Uvala	44.92	15.50	978–1029	7.90	1963.50	72.00	10	161	193 (±98)	44.8 (±30.7)
Croatia	CRO	Cudinka	44.88	15.53	1096–1152	6.95	2019.80	70.78	10	118	251 (±106)	43.2 (±34.7)
Croatia	CRO	Rjecica	44.86	15.62	652–734	9.35	1755.20	70.19	14	213	202 (±134)	46.6 (±30)
Croatia	CRO	Smrceve Doline	44.77	14.98	1361–1534	5.76	2293.80	62.83	22	846	213 (±49)	28.4 (±13.1)
Croatia	CRO	Ramino Korito	44.49	15.19	821–984	7.84	1945.40	43.86	16	407	220 (±68)	46 (±19.9)
Bosnia	BOS	Perucica	43.31	18.70	1057–1449	4.67	1521.80	66.67	47	605	139 (±153)	29.5 (±38)
Bulgaria	BUL	Boatin	42.79	24.27	1236–1449	5.79	1023.10	50.04	14	304	221 (±92)	44.2 (±32.2)
Bulgaria	BUL	Steneto	42.74	24.69	1246–1482	5.82	1159.20	46.21	28	564	205 (±121)	50.7 (±33.3)
Albania	ALB	Curraj i Eperm	42.36	19.86	1006–1278	8.65	2822.30	41.95	14	361	116 (±43)	38.6 (±21.9)
Albania	ALB	Lumi i Gashit	42.49	20.04	1209–1688	6.93	2578.00	37.33	14	368	196 (±29)	45 (±26.5)

*Note:* Descriptive characteristics of forest stands and climatic conditions under climate warming (1980–2020). Columns denote region (Country), regional acronym (Code), forest stand (Stand), latitude (Lat), longitude (Lon), elevation (m.a.s.l), average annual temperature (MAT; °C), precipitation totals (MAP; mm/year), and summer climatic water balance (CWB summer; mm/year), total number of plots (N plot) and trees (N tree), and median tree age (Age; year) and diameter at breast height (DBH; cm). Numeric values in brackets are interquartile ranges (IQR). Sites are ordered by latitude (southward) and along the summer moisture (CWB summer) gradient under climate warming (since the year 1980).

### 2.2 Data Collection and Processing

Field data collection followed protocols established within the REMOTE network to ensure consistency and cross‐study comparability. In short, sampling plots were established using a stratified random design to capture the spatial variability across European beech primary forests. A 10‐ha grid was overlaid across each forest area, and random points were generated within each 3.4‐ha grid cell. In case of the unsuitability of a randomly generated point due to difficult terrain (e.g., rocky terrain, steep slopes, etc.), an alternative point was randomly generated within a grid cell. This design established a hierarchical sampling structure, with plots nested within stands and stands nested within regions, thereby maximizing the representativeness of ecological gradients across biogeographic regions. Pairs of circular sampling plots (1500 m^2^) were established 40 m in each direction from the randomly generated points along the slope contours. Within each sampling plot, topography (i.e., slope, aspect, and elevation) and biometric data (i.e., species, tree position within the plot, canopy, and living status) were recorded. In total, we used dendrometric and structural data from 530 sampling plots nested within 33 distinct forest stands (Table 1).

Increment cores were obtained from all trees with DBH ≥ 10 cm within the inner 200 m^2^ circular subplot at each sampling plot. In addition, trees with DBH ≥ 20 cm and approximately 25% of non‐suppressed trees with DBH ≥ 10 cm were cored outside of the inner subplot. All cores were collected at 1 m stem height perpendicularly to the dominant terrain slope to avoid reaction wood (for a more detailed description of sampling protocols, see Janda et al. 2025; Marchand et al. 2023; Pavlin et al. 2024). Cores were processed following the established protocols by Stokes and Smiley (1996), which involved air‐drying, mounting on wooden boards, and sanding with progressively finer‐grit sanding paper until annual growth rings were clearly demarcated. Annual ring‐width measurements (RW) were obtained using the LinTab stereomicroscopes with a sliding measuring stage and integrated TSAP‐WinTM measuring software, and were visually cross‐dated based on the extreme growth year approach (Yamaguchi 1991). Dating quality was validated using the CDendro software (v. 9.6; Larsson 2015). After excluding cores that could not be reliably cross dated, and those missing more than 30 mm or 20 years to the pith, we retained 10,860 cores for further analysis (Begović et al. 2026).

### 2.3 Data Standardization

Two growth metrics were derived from measured RWs: detrended ring width indices (RWI) for climate response analysis, and basal area increments (BAI) for growth trend analysis.

Dimensionless RWIs delineate the impact of monthly‐to‐seasonal climatic variation on tree growth, while minimizing the effects of non‐climatic and ontogenetic trends (Cook and Peters 1997). Individual RW time series were detrended using a cubic smoothing spline with a 50% frequency response cutoff at 10 years to isolate the climate signal and preserve high‐frequency variability. RWIs were produced as ratios between observed and modelled values. Residual chronologies were obtained by applying an autoregressive model to detrended chronologies in each year (Cook and Pederson 2011) and then aggregated as Tukey's bi‐weight robust means into four distinct demographic groups to capture potential physiological contrasts in ontogeny‐related traits: large old (*l_o*; 75th age & DBH quantile), large young (*l_y*; 25th age quantile, 75th DBH quantile), small old (*s_o*; 75th age and 25th DBH quantile), and small young trees (*s_y*; 25th age and DBH quantile). Mean inter‐series correlation (*rbar*) and expressed population signal (*EPS* > 0.80) statistics were used to validate the robustness of residual chronologies (Wigley et al. 1984).

BAI directly measures cross‐sectional area variations based on DBH and provides an accurate representation of wood production and biomass variability (Visser 1995; West 2015). Individual BAI time series were derived from reconstructed stem diameters using the formula for circular stem cross‐section. To minimize the impacts of age‐ and size‐related trends on growth variability with increasing tree size (Briffa and Melvin 2011), annual BAIs were detrended using a mixed‐effects modelling approach (Klesse and Bigler 2025). Individual BAI time series were log‐transformed to stabilize the variance and normalize the distribution. Then, linear mixed‐effects models were used to predict log‐transformed BAI as a function of the previous year's log‐transformed DBH, with stand‐level random intercepts and slopes for log‐transformed DBH and tree‐level random intercepts (trees nested within stands) to account for between‐site variability (Bates et al. 2015). The predicted log‐transformed BAIs were back‐transformed to original measurement units (cm^2^/year), and size‐detrended BAI (BAI_sdt_) chronologies were obtained by dividing the observed with predicted BAI. Tukey's bi‐weight robust mean was used to yield stand and regional BAI_sdt_ chronologies of non‐overlapping age (< 100, 100–199, 200–299, and 300+ years) and size (10–39, 40–60, and 60+ cm) classes. Stand‐level BAI_sdt_ chronologies generally showed a strong high‐frequency agreement with observed BAI chronologies since the mid‐20th century (Figure S4). Minimum sample size threshold was established using the power analysis with Bonferroni correction (Welch's *t*‐test, *α* = 0.05, power = 0.80) and Cohen's effect sizes to ensure robust sample replication across regions and demographic classes.

### 2.4 Data Analysis

To address the main research questions (Q1–Q3), we combined dendrochronological with correlation analysis, non‐parametric statistical approaches, and hierarchical modelling. We provide a conceptual summary of the analytical workflow in Table 2, and describe each statistical approach in detail below.

**TABLE 2:** Summary of the study methodological framework.

Research question	Statistical method	Response variable	Explanatory variable	Hierarchical level	Output
Q1: Which climatic factors most strongly control radial growth of different demographic groups under recent climate warming, and do demographic groups respond uniformly to severe summer droughts across ecological gradients?	Monthly bootstrapped Pearson's correlations Random‐effects meta‐analysis with Fisher's *z* transformation	Residual ring‐width index chronologies (RWI)	Monthly and seasonal climate variables: minimum temperature (T_min_), maximum temperature (T_max_), climatic water balance (CWB = precipitation − potential evapotranspiration)	Demographic group → Stands/Regions	Identification of dominant climate drivers of radial growth and comparison across demographic groups over the biogeographic gradient
	Double‐bootstrapped superposed epoch analysis (SEA)	RWI response to drought events	Summer climatic water balance (CWB_summer)	Demographic group × biogeographic gradient type	Quantification of demographic differences in growth response to severe droughts
	Moving‐window Pearson's correlation analysis	Moving window correlations (*r*)	Climatic variables as above	Region Demographic group × biogeographic gradient type	Detection of temporal shifts in regional and demographic group climate sensitivity under warming
Q2: Do demographic groups exhibit divergent long‐term growth trends under recent climate change and, if so, do these divergences vary across ecological gradients?	Regression with Sen's slope (growth trend) with adjusted Mann‐Kendall test (significance)	Size‐detrended basal area increment (BAIsdt) chronologies	Calendar year Demographic class (age × size) Regional (climatic) gradients	Tree → Demographic group → Region	Identification of directional growth trends across demographic groups and regions
	ART ANOVA and Chi‐squared tests	Distribution of positive vs. negative growth trends	Individual‐tree Sen's slope estimates	Tree → Stand → Region	Evaluation of demographic differences in growth trend direction
	Univariate regression models with heteroscedasticity‐consistent errors	Association between growth trends and climate	Demographic group median Sen's slope estimates Climate variables (long‐term average and Δ from 1940–1979 to 1980–2020)	Stand → Network	Identification of key associations between climatic factors and demographic growth trends
Q3: How do demographic variability in growth dynamics and forest structure interact with climate variability to shape forest ecosystem productivity under climate warming?	Aggregation and bootstrapping procedures	Stand‐level growth trend contribution (y, m^2^ ha^−1^ yr.^−1^)	Tree‐level Sen's slope trends Cumulative basal area (m^2^ ha^−1^ yr.^−1^) + tree density (N ha^−1^) within each demographic class	Tree → Stand × Demographic group → Region/Biogeographic gradient type/Network	Assessment of how demographic growth dynamics modulate within‐ and cross‐regional productivity trends under climate warming
	Hierarchical mixed‐effects model (LMM)		Fixed Effects: Demographic class (age × size) Trend direction (+/−) Biogeographic gradient type Random effects: Stands nested within regions		
	Generalized linear mixed‐effects model (GLMM)	Annual stand‐level productivity (BAI, m^2^ ha^−1^ yr.^−1^)	Fixed effects: Demographic productivity index (DPI) Stand basal area (m^2^ ha^−1^) Tree density (trees ha^−1^) Tree age and size inequality (Gini coefficient) Principal component of prior and current year climate (PC scores) Cosine‐transformed aspect (untiless) Terrain slope (degrees) Biogeographic gradient type Random effects: Stands nested withing regions Calendar year	Stand/Region	Quantification of relative contributions of climate, structure, and demographic variability to predicted forest productivity over the period 1980–2020.

*Note:* The table summarizes the analytical workflow of the main statistical approaches and expected outputs linked to individual study research questions. The response and explanatory variables with their associated units, the hierarchical levels of data analysis, and all relevant abbreviations and acronyms are briefly described.

#### 2.4.1 Climate Response Analysis

Bootstrapped Pearson correlation coefficients were used to assess the relationships between RWI stand chronologies and climate data over an 18‐month period, spanning from June of the year preceding ring formation to October of the current ring‐formation year. Monthly climate variables were also aggregated to describe seasonal climatic conditions (i.e., “*WINTER*” from previous year December to current year February, “*SPRING*” as March to May, “*SUMMER*” as June to August, “*GS*” as growing season, i.e., April to September, and “*p_summer*” as prior year June to August). All climatic parameters were detrended “like‐for‐like” using a cubic smoothing spline (50% frequency cutoff at 10 years) to reduce the potential impacts of climate trends (Ols et al. 2023). Climate response analysis was performed on residual RWI stand (*n* = 33) and demographic group chronologies (*n* = 4) using *treeclim* R package (Zang and Biondi 2015) across two successive time periods, before (1940–1979) and after (1980–2020) the onset of accelerated warming, and over the full period (1940–2020). Stand‐ and group‐level correlations were aggregated at the regional level using inverse‐variance weighting and Fisher's z‐transformation with random‐effects meta‐analysis to account for spatial heterogeneity (Hartung et al. 2008). Bootstrapped confidence intervals were used for robust uncertainty estimates.

Additionally, we evaluated spatial patterns in dominant climatic modes using PCA based on a correlation matrix of bootstrapped Pearson's correlations between regional RWI residual chronologies and selected climate variables and assessed the temporal stability of key climate‐growth relationships using the moving window correlation function (Biondi 1997). To quantify intraspecific differences in growth response to severe droughts, we employed a modified double‐bootstrap superposed epoch analysis approach (SEA; Rao et al. 2019). Drought events were selected independently of the degree of RW reduction, as years with a 1.5 standard deviation below the mean of summer CWB (Kannenberg et al. 2020) recorded in more than 90% of forest stands. We used a 7‐year window length, spanning 3 years pre‐ and post‐drought to avoid overlapping drought events, and aggregated epochal means along the ecological gradient at demographic group level using the same hierarchical meta‐analytic approach as in the climate response analysis.

#### 2.4.2 Growth Trend Analysis

Regional and class‐based BAI_sdt_ chronologies were linearly regressed against calendar year using Sen's slope coefficient to quantify directional trends in age‐ and size‐specific chronologies before (1940–1979) and after (1980–2020) the onset of accelerated climate warming. This periodization ensured comparability between successive time periods while excluding juvenile growth effects in the youngest age class (< 100 years). Trend significance was quantified using a variance‐corrected Mann‐Kendall test from the *modifiedmk* R package (Patakamuri and O'Brien 2021) to control for heteroscedasticity and type I error. Regional differences in growth trend strength (i.e., Sen's slopes) and distribution of trees with positive versus negative growth trends under climate warming were assessed using aligned rank transform (ART) ANOVA, to account for non‐normal distributions between tree cohorts, with the Benjamini‐Hochberg (BH; Benjamini and Hochberg 1995) post hoc and the Chi‐Squared test, respectively.

We employed a series of univariate regression models to assess whether spatial patterns in recent growth trends (i.e., mean Sen's slope values) are associated with long‐term average climatic conditions and changes in seasonal climatic drivers. Changes in seasonal climate variables were quantified as the difference in average climatic values from the pre‐warming (1940–1979) to the post‐warming period (1980–2020). Climatic predictors were analysed separately to avoid multicollinearity and to isolate the strength of each association between demographic classes and dominant climatic drivers identified by the climate response analyses (Table S1). Models were fitted with a heteroscedasticity‐consistent standard error structure to account for unequal variances between demographic classes and stands. Significance values were adjusted by the BH false discovery rate.

We also quantified contributions of individual age and size classes to regional productivity trends to assess which individual demographic classes are buffering or amplifying beech ecosystem productivity trends under climate warming. Tree‐level Sen's slope values were weighted by cumulative basal area and scaled by tree density (N ha^−1^) within each demographic class to derive stand‐level class productivity contributions (m^2^ ha^−1^ year^−1^). Positive and negative contributions, based on the direction of individual tree growth trends, were aggregated within each region to yield regional net productivity trends (*y*_net_). To quantify whether demographic class contributions differed systematically across biogeographic gradients under climate warming, we fitted a hierarchical mixed‐effects model with demographic class, trend direction (positive versus negative), and biogeographic gradient type (summer‐mesic versus summer‐xeric) as fixed effects, and stands nested within regions as random intercepts. Estimated marginal means were compared within each demographic class and trend direction using BH‐adjusted pairwise contrasts.

#### 2.4.3 Modelling Framework

Finally, we employed a mixed‐effects modelling framework to quantify the relative effects of forest structure, climatic variation, topography, and demographic variability in growth dynamics on forest productivity under climate warming.

We modelled log‐transformed annual stand basal area increment (BAI; m^2^ ha^−1^ yr.^−1^) using a generalized linear mixed‐effects model (GLMM; Equation 2) with a Gaussian distribution. Stand structural predictors included tree density (N ha^−1^), stand basal area (m^2^ ha^−1^ yr.^−1^), and Gini coefficients of tree age and size (DBH) variance calculated from the empirical distribution of individual tree age and DBH values within each stand using the *DescTools* R package (Signorell et al. 2025). Climatic predictors were represented by linear and quadratic polynomials of two principal components, denoting previous summer (PC1) and current year spring and summer annual temperature (T_max_, T_min_) and moisture (CWB) conditions (PC2), respectively. Physiographic covariates (i.e., slope and northness as a cosine‐transformed aspect) were also included in the model. We also integrated a demographic productivity index (DPI) to account for disproportionate contributions of demographic groups with positive versus negative trends to forest productivity during the warming period (1980–2020). The index was calculated as the cumulative annual basal area share of each demographic class, and weighted by the median class‐specific Sen's slope trend (Equation 1):

(1) $${\mathrm{DPI}}_{s,t}=\sum_{g}\;{\mathrm{BA}}_{s,t,c}\times \beta_{s,c}$$

where *w* denotes total basal area share in each stand (*s*), demographic class (*c*) and year (*t*), and *β* represents the median class‐level Sen slope estimate over the period 1980–2020. Positive index values depict stands dominated by demographic classes with positive growth trends, whereas negative index values denote the prevalence of the declining classes under recent warming. We also included pairwise interactions between climate covariates, demographic productivity index, and the biogeographic gradients to disentangle the impacts of climatic conditions on demographic contributions to forest stand productivity. Random intercepts were fitted for stands nested within regions to account for the hierarchical structure of our dataset, and calendar year to account for shared interannual variability across the study network. We applied model weights using stand‐level replication to reduce the impact of disproportionately abundant stands on model predictions and error estimation, so the final model structure was:

(2) $$y_{s,t}=\beta_{0}+\beta \;\left[\left(C_{s,t}\right)+g\;\left(S_{v}\right)+{\mathrm{DPI}}_{s,t}+{\mathrm{DPI}}_{s,t}\times \left(C_{s,t}\right)\right]+u_{\text{country}}+u_{\text{stand}}+u_{\text{year}}+\varepsilon$$

where *β*_0_ represents the intercept of expected log‐transformed basal area increment (*y*_*s,t*_), *S* are stand structural and topographic predictors for each stand (*s*), *f*(*C*) are orthogonal polynomials of principal climate components for each stand and year (*t*), DPI_*s,t*_ *×* C_*s,t*_ are climate and DPI interactions, *u* are random intercepts accounting for the hierarchical structure of the dataset, and *ε* denotes unexplained residual variation.

All continuous predictors were centered and scaled (mean = 0, SD = 1) to facilitate direct comparisons of effect sizes across predictors. Multicollinearity between predictors was tested using the variance inflation factors (VIFs; Dormann et al. 2013) and was not a significant issue (VIF < 5). The most parsimonious model was determined by the “leave‐one‐out” likelihood‐ratio approach (*p <* 0.05; Johnson and Omland 2004), in which individual predictors are removed and reduced models compared using ΔAIC (Akaike Information Criteria; Akaike 1974; Zuur et al. 2009) as the indicator of parsimony and likelihood (Burnham and Anderson 2004). Same approach was repeated for the random effect structure. Additional model validation was performed by comparing models with and without demographic productivity index and their climate interactions to estimate the contribution of each set of predictors to modelled stand productivity estimates. The final model was refitted with the most relevant predictors using restricted maximum likelihood (REML). Visual and statistical diagnostics of model residuals, including heteroscedasticity, zero‐inflation, overdispersion, and temporal autocorrelation, revealed no significant modelling issues (Figure S8). Model performance was based on the proportion of total variance explained by fixed effects only (i.e., marginal *R* ^2^) and fixed and random effects (i.e., conditional *R* ^2^) following Nakagawa and Schielzeth (2013). In addition, we quantified unique explanatory contributions of each individual predictor using semi‐partial R^2^ (Jaeger et al. 2017).

Data analysis and model fitting were performed using the R software (R Core Team 2024) packages: *tidyr* for data manipulation (Wickham and Girlich 2024), *glmmTMB* for model fitting (Brooks et al. 2025), *performance* (Lüdecke et al. 2021), and *DHARMa* (Hartig, 2015/Hartig 2022) for model diagnostics, *MuMIn* (Bartoń 2013) for model coefficient calculations, and *ggplot2* (Wickham 2016) for graphical visualization.

## 3 Results

### 3.1 Regional and Demographic Climate‐Growth Relationships

Based on the hierarchical meta‐analytical synthesis of regional climate correlations, previous summer maximum temperature (T_max_p_summer_) and average climatic water balance (CWB__p_summer_) exhibited the strongest and most consistent correlations with RWI variation across the network over all three time periods (Table S1, Figure S5; *r*_abs_ = 0.29–0.33, *p <* 0.01), although current spring (T_min_SPRING_ and CWB__SPRING_) and summer climate (T_max_SUMMER_) became strong regional co‐factors over recent decades. Although precipitation exhibited comparable correlations with RWI variation, it was dropped from further analysis as CWB demonstrated broader cross‐regional correlations. Ordination analysis revealed a spatial gradient in growth sensitivity to seasonal climatic variation under climate warming (Figure S6a), between more mesic northwestern regions—showing higher sensitivity to T_min_SPRING_, CWB__SPRING_ and T_max_SUMMER_ (positive PC1 scores)—and more xeric southeastern regions—with stronger response to T_max_p_summer_ and CWB__p___summer_ (negative PC1 and PC2 scores). Although regional growth correlations with seasonal climatic drivers temporally varied in strength and significance, sensitivity to T_max_p_summer_ increased across the network over recent decades (Figure S6b).

**FIGURE 2:**
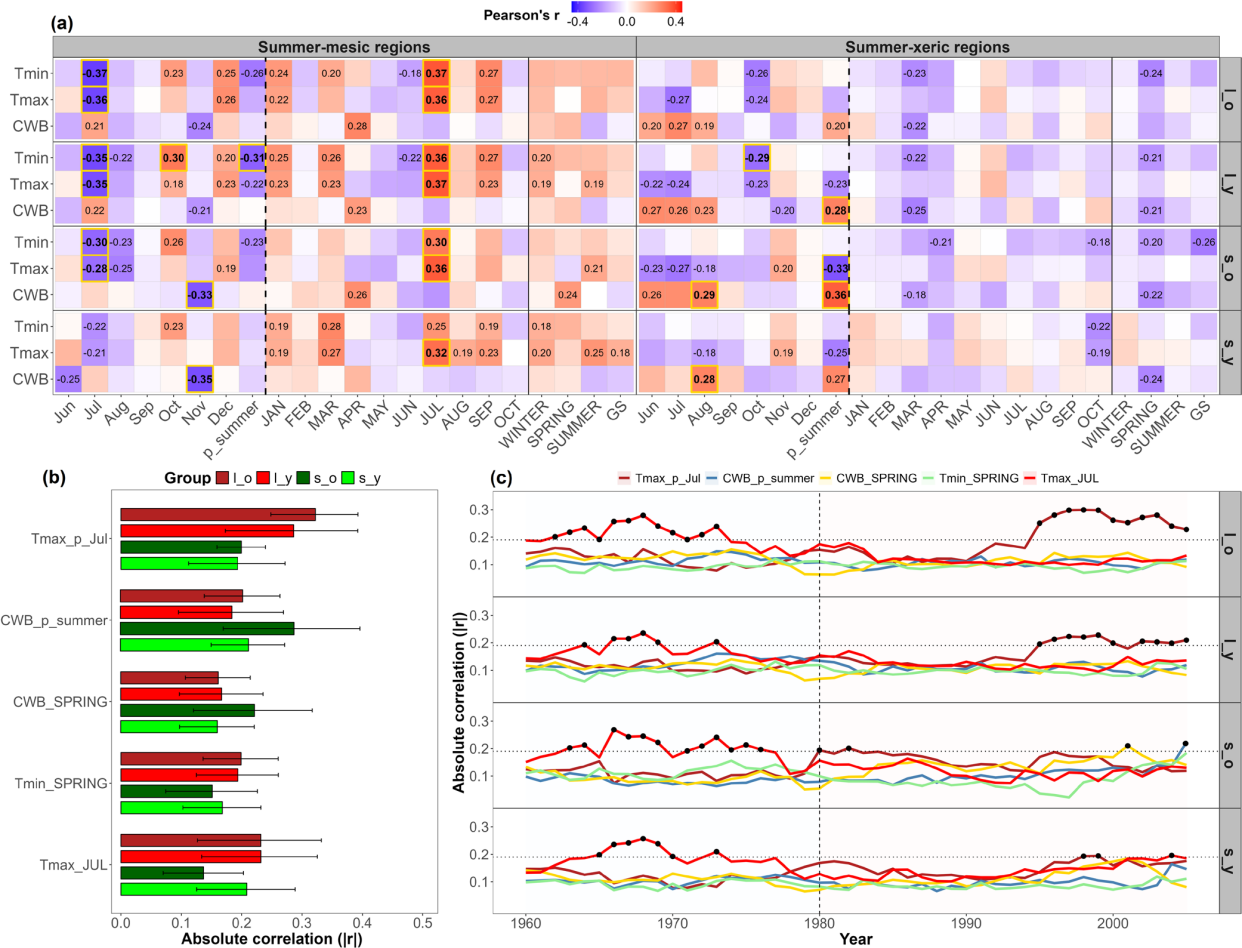
Demographic variability in climate‐growth sensitivity under climate warming. (a) Heatmap of Pearson's correlations between residual ring‐width index (RWI) chronologies of demographic groups and monthly/seasonal temperature (T_min_ and T_msc_) and climatic water balance (CWB) over the period 1980–2020. Significant correlations (at *p <* 0.05) are denoted by *r* coefficients. Tiles with the strongest correlations are yellow outlined and with bolded *r* values. Capital letters denote months and seasons of the current growing year, otherwise are shown months of the year prior to tree ring formation. Vertical lines separate aggregated previous (dashed) and current (full) months and seasonal windows. (b) Bar plots of mean absolute correlations (|*r*|) with bootstrapped 95% confidence intervals and (c) temporal variation in absolute correlations between RWI group chronologies and key climatic factors. The vertical dashed line marks the start of the warming period, while the horizontal dashed line denotes the significant effect‐size reference based on the random‐effects meta‐analytic model. Acronyms denote demographic groups as *l_o* (large old), *l_y* (large young), *s_o* (small old), *s_y* (small young).

Demographic groups in summer‐mesic regions exhibited more pronounced responses to current (*r* = 0.25–0.37) and previous July T_max_ (*r* = −0.21 to −0.37) variation, whereas the strongest relationships in summer‐xeric regions were with CWB__p___summer_ (*r* = −0.27 to −0.36), particularly in large young (*l_y*) and small old (*s_o*) trees (Figure 2a). Notably, beech also showed a significant positive relationship with previous autumn temperatures (T_min_ October) and a negative relationship with previous November CWB (*s_o* and *s_y*) in summer‐mesic regions. Small trees showed higher absolute correlations with prior summer and current spring CWB, whereas large trees showed stronger sensitivity to prior and current year July T_max_ variation (Figure 2b). In general, large trees showed significant increasing sensitivity to previous July T_max_, whereas small trees demonstrated weak (*r*_abs_ ~0.1) but notable rising sensitivity to CWB__p___summer_ (*s_o*), CWB__SPRING_ (*s_o*), and July T_max_ (*s_y*) over recent decades (Figure 2c).

**FIGURE 3:**
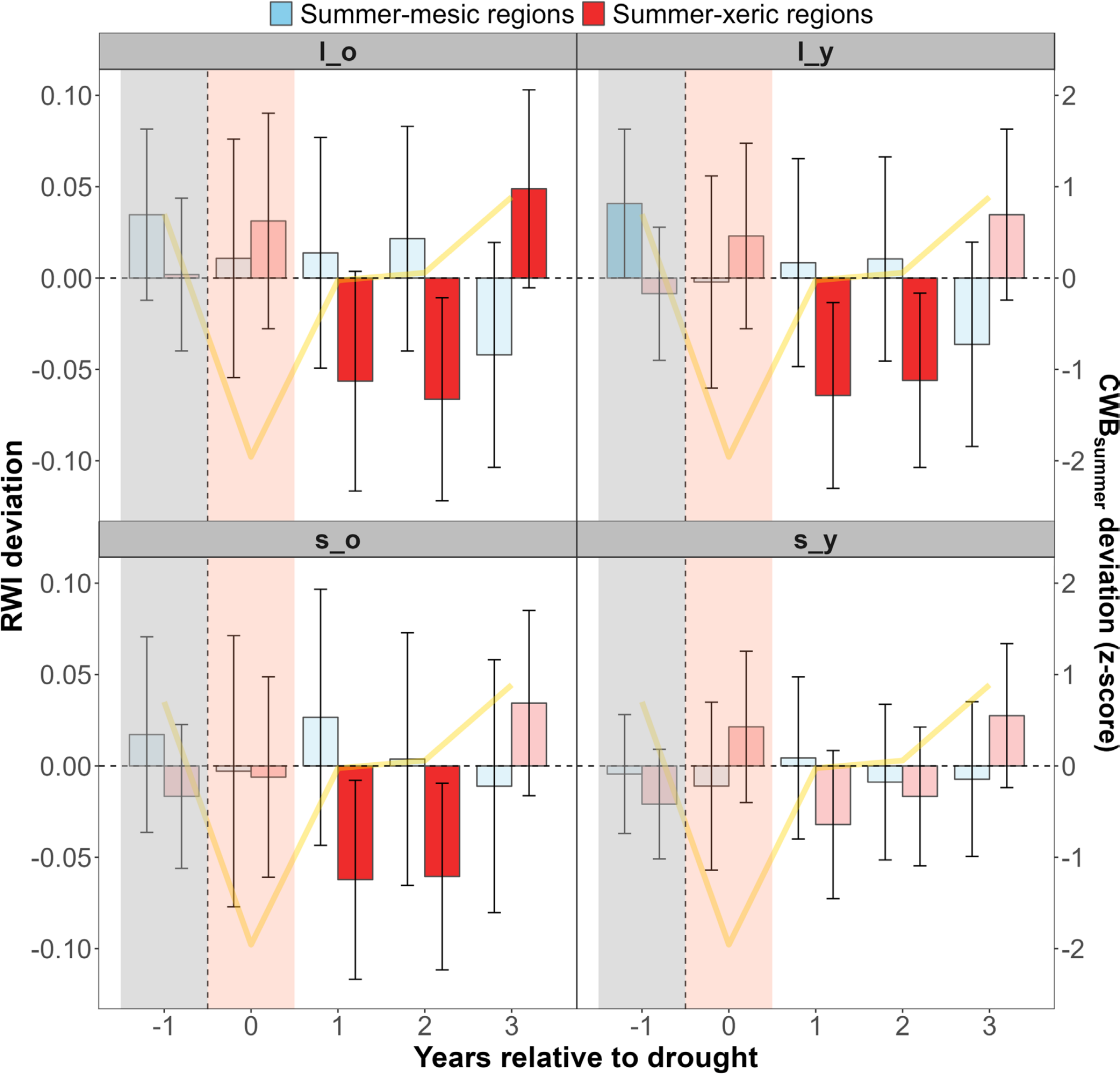
Demographic growth responses to severe summer droughts. Superposed epoch analysis (SEA) of mean residual ring‐width index (RWI) deviations before and after severe summer droughts across demographic groups and biogeographic gradients. Unsaturated bars with bootstrapped 95% confidence intervals denote significant pooled SEA effect sizes estimated using the multilevel random‐effects model. Yellow lines show standardized deviations (z‐scored) of summer climatic water balance (CWB__SUMMER_). Positive and negative bars indicate above‐ or below‐average RWI deviations relative to the baseline. Pre‐drought period was reduced to a single (−1) year for easier visualization. Acronyms denote demographic groups as *l_o* (large old), *l_y* (large young), *s_o* (small old), *s_y* (small young).

SEA revealed distinct drought response patterns across the climatic gradient (Figure 3). Summer‐xeric regions exhibited strong negative RWI deviations in the subsequent years across demographic groups (*β* ~−0.06, *p* < 0.05), although small young (*s_y*) trees showed weaker and less consistent post‐drought deviations compared to other demographic groups. In contrast, summer‐mesic regions showed weak and generally non‐significant RWI deviations during and after severe droughts, although substantial between‐stand variability in drought response was observed in the northwestern (Slovakian) regions (i.e., W SLO, E SLO, C SLO; Figure S6c).

### 3.2 Regional and Demographic Growth Dynamics

**FIGURE 4:**
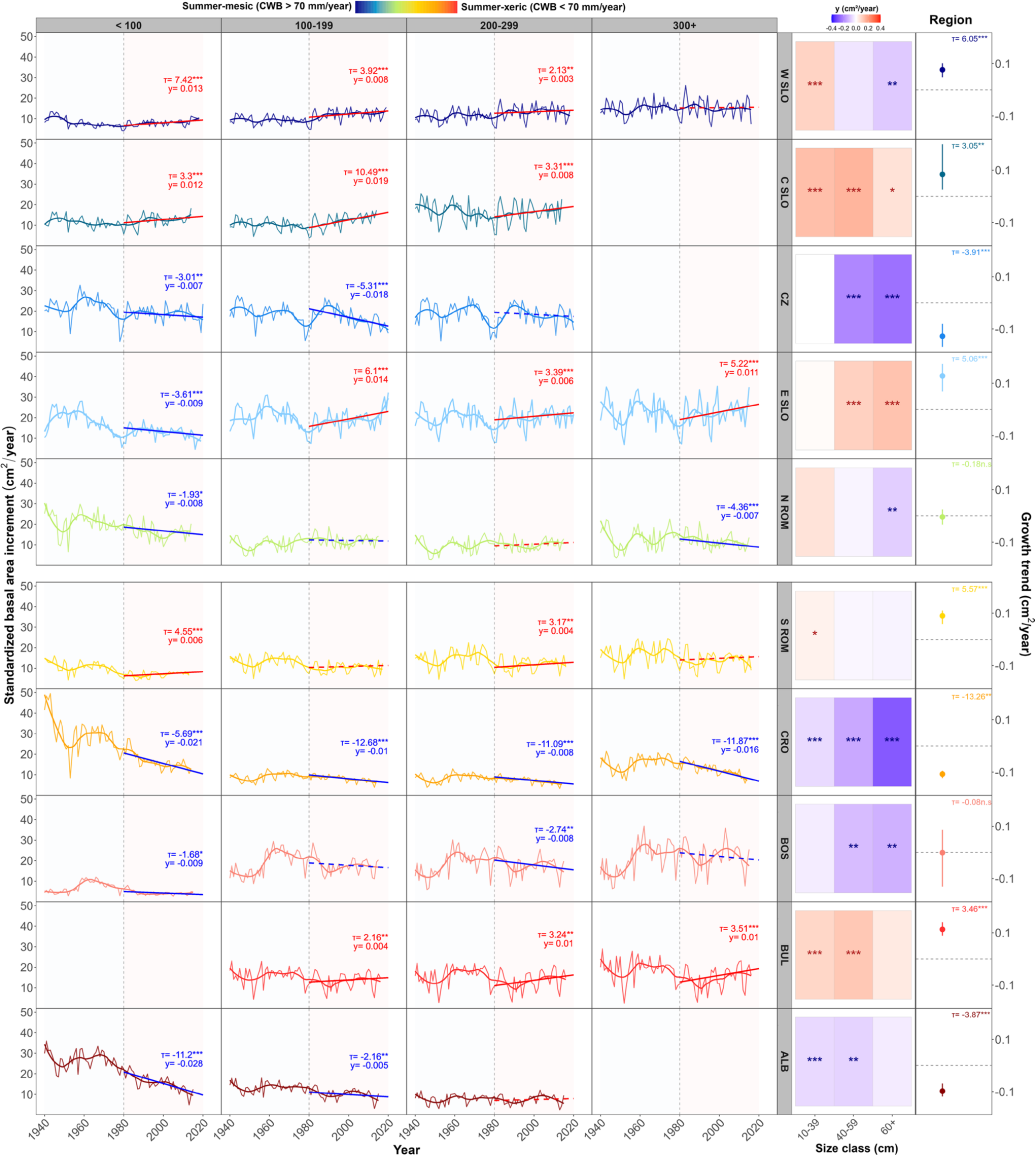
Long‐term regional and demographic growth dynamics. Standardized basal area increment chronologies (BAI_sdt_) of individual age classes across study regions. Smoothed curves are plotted as generalized additive models with cubic regression splines (*gam*) for easier visual interpretation. Linear regression lines with Sen's slope estimates (y, cm^2^ year^−1^) and Mann‐Kendall's adjusted tau metric (*τ*) denote significant positive (red) and negative (blue) growth trends (*p <* 0.1 (*), < 0.05 (**), < 0.001 (***)), while dashed lines show non‐significant trends. Bar plots and the point‐range plots (right) with asterisks depict significant growth trends of individual size classes and regional aggregates under climate warming (i.e., 1980–2020), respectively. Points represent y (cm^2^ year^−1^), vertical lines denote bootstrapped 95% confidence intervals, and accompanying *τ* values are shown with significance levels. The vertical dashed line marks the start of the warming period. Regional chronologies are arranged along a summer moisture (CWB_summer_) gradient from mesic to xeric. Regional acronyms denote regions as defined in Table 1.

Growth trend analysis revealed distinct regional divergences under climate warming (Figure 4), with strong negative trends in the Dinaric Mts. (e.g., Croatia/CRO and Albania/ALB) and the Jizera Mts. (i.e., Czechia/CZ), and significant positive trends in the Western Carpathians (i.e., Slovakia/SLO; W SLO, E SLO, and C SLO), the southern Carpathians (i.e., southern Romania/S ROM), and the Balkan Mts. (i.e., Bulgaria/BUL).

Significant growth declines were primarily driven by mid‐ and large‐sized young trees (< 200 years, > 40 cm), except in CRO, where significant negative trends were found across all demographic groups, and N ROM, where the smallest and oldest trees exhibited the strongest declines. The magnitude and timing of growth declines varied between regions and demographic groups (*y* ~−0.28 to ~−0.04 cm^2^/year, *p <* 0.05), with exceptionally steep declines in the Dinaric Mts. (i.e., CRO, BOS, ALB) starting around the 1960s and in the Jizera Mts. starting from the 1990s (i.e., CZ; *y* ~−0.26 cm^2^/year). In contrast, positive growth trends were driven by small to mid‐sized trees of various age groups in W SLO and C SLO, large young and mid‐aged trees in E SLO, and small and mid‐sized old trees in BUL. Notably, aggregated demographic growth divergences did not translate into significant mean regional growth trends in N ROM and BOS.

**FIGURE 5:**
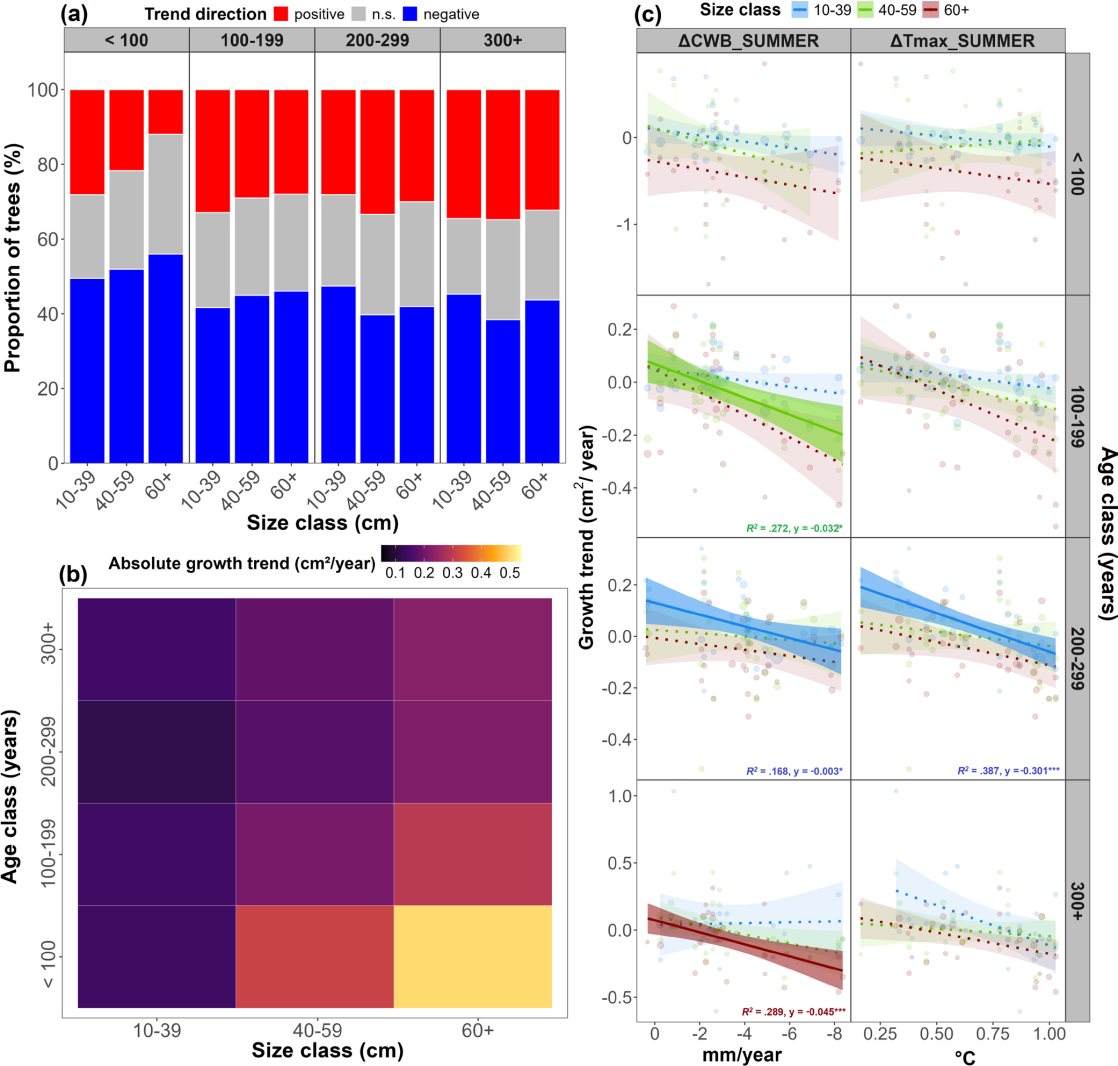
Demographic variability in growth dynamics under climate warming. (a) Bar plots of tree proportions with positive (red), negative (blue) and non‐significant (gray) growth trends across demographic classes over the period 1980–2020. (b) Heatmap of mean absolute Sen's slope values for individual demographic classes. (c) Sen's slope values modelled in relation to change in summer climatic conditions (CWB and T_max_). Solid regression lines indicate statistically significant relationships at different significance levels (*p*_adj_ < 0.1 (*), < 0.001 (***)) across individual demographic classes. Shaded areas around regression lines represent 95% confidence intervals. Growth trends are plotted on different scales across age classes for easier visualization.

Most regions and demographic groups exhibited significantly lower productivity under climate warming, with only mid‐aged small trees in mesic regions showing significantly higher average BAI_sdt_ compared with the preceding time period (Figure S7a). However, there was a large within‐regional variability in strength and direction of recent BAI_sdt_ trends (Figure S7b), with an almost equal proportion of trees with positive versus negative growth trends across the network, except in C SLO—where the proportion of trees with positive trends was twofold greater—and the Dinaric regions, where significantly more trees exhibited growth declines (Figure S7c). Overall, more trees exhibited significant negative (45.1%) than positive (30.4%) growth trends across the network (Figure 5a), with young and mid‐aged large trees (< 200 years, > 40 cm) exhibiting the strongest absolute growth trends under climate warming (*y*_*median*_ > 0.37 cm^2^/year; Figure 5b, Figure S7d).

The magnitude and direction of recent growth trends were most strongly associated with changes in maximum summer temperature (ΔT_max_SUMMER_) and average summer climatic water balance (ΔCWB__SUMMER_) from pre‐warming to post‐warming period across regions and demographic classes (Figure 5c). Rising summer heat (*β* = −0.24, *p* < 0.001) and declining moisture availability (*β* = −0.03, *p* < 0.001) markedly intensified negative growth trends consistently across demographic groups under climate warming, although the response magnitude to ΔT_max_SUMMER_ and ΔCWB__SUMMER_ was considerably stronger in small mid‐aged trees, and small mid‐aged and large old trees, respectively.

### 3.3 Regional Productivity Trends and Drivers of Ecosystem Productivity

**FIGURE 6:**
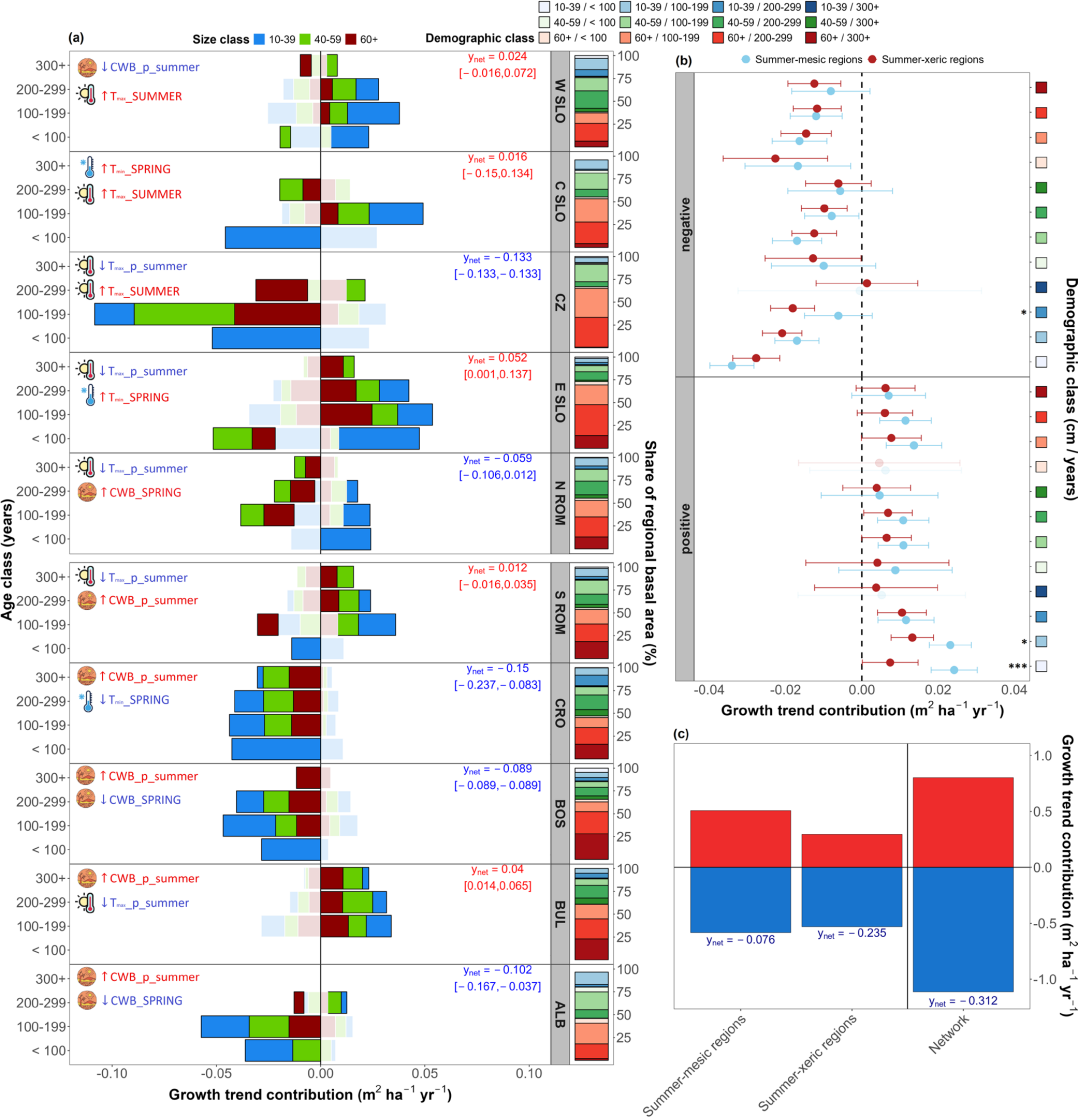
Regional and demographic productivity trends under climate warming. (a) Horizontal stacked bar plots of demographic class mean contributions to regional productivity trends (*y*_netl_; m^2^ ha^−1^ year^−1^) over the period 1980–2020. Positive and negative contributions are separately stacked, and regional net productivity estimates are shown with bootstrapped 95% confidence intervals. Color saturation indicates the relative dominance of the within‐class trend direction (unsaturated bars denote stronger contributions). Adjacent vertical stacked bar plots show the mean regional basal area share (%) of individual demographic classes. Icons with text denote positive (red, upwards arrow) and negative (blue, downwards arrow) radial growth relationships with key seasonal climate drivers identified in the climate‐response analysis across regions (see Figure S7a). Regions are arranged along a summer moisture (CWB) gradient from mesic to xeric. Regional acronyms denote regions as defined in Table 1. (b) Bootstrapped marginal means with error bars (±95% CI) from the hierarchical mixed‐effects model show demographic class contributions by trend direction and biogeographic gradient type. Asterisks denote significant within‐class differences. (c) Bar plots of separately aggregated productivity gains versus losses for summer‐mesic, summer‐xeric regions and the entire study network.

The strongest and most consistent negative productivity trends were observed in the Dinaric Mts. (*y*_net_ ~−0.114 m^2^ ha^−1^ yr.^−1^; Figure 6a), where declines were primarily driven by small (< 40 cm) young (ALB; < 200 years) and mid‐aged trees (BOS; 100–299 years), and large mid‐aged and old trees (CRO; > 200 years, > 40 cm). Outside the Dinaric regions, negative net productivity trends were also evident in CZ (*y*_net_ ~−0.133 m^2^ ha^−1^ yr.^−1^) and N ROM (*y*_net_ ~−0.059 m^2^ ha^−1^ yr.^−1^), and were mainly driven by declines in mid‐sized and large young (> 40 cm, < 200 years) and large mid‐aged and old trees (> 200 years), respectively.

In contrast, positive productivity trends noted in the northwestern regions were driven by gains in small and mid‐sized trees (< 60 cm) from young and mid‐aged classes (W SLO, C SLO; < 200 years), as well as large mid‐aged trees (E SLO; > 60 cm). Notably, positive net productivity was also observed in two southeastern (xeric) regions (*y*_net_ S ROM = 0.012; *y*_net_ BUL = 0.04 m^2^ ha^−1^ yr.^−1^), where gains in small and mid‐sized young and mid‐aged trees (S ROM; < 200 years), and large old trees (BUL; > 200 years, > 40 cm), outweighed declines in other demographic classes. On average, positive demographic contributions to regional productivity trends were higher in summer‐mesic regions (Figure 5b), particularly in small young trees (10–39 cm and < 200 years; *p* < 0.05), whereas negative contributions were pervasive across ecological gradients, but significantly stronger in small mid‐aged trees from summer‐xeric regions (10–39 cm and 200–299 years). Overall, total productivity losses outweighed gains across biogeographic gradients (*y*_net_ mesic = −0.076 m^2^ ha^−1^ yr.^−1^, *y*_net_ xeric = −0.235 m^2^ ha^−1^ yr.^−1^) and over the whole study area (*y*_net_ network = −0.312 m^2^ ha^−1^ yr.^−1^) under recent warming (Figure 5c).

**FIGURE 7:**
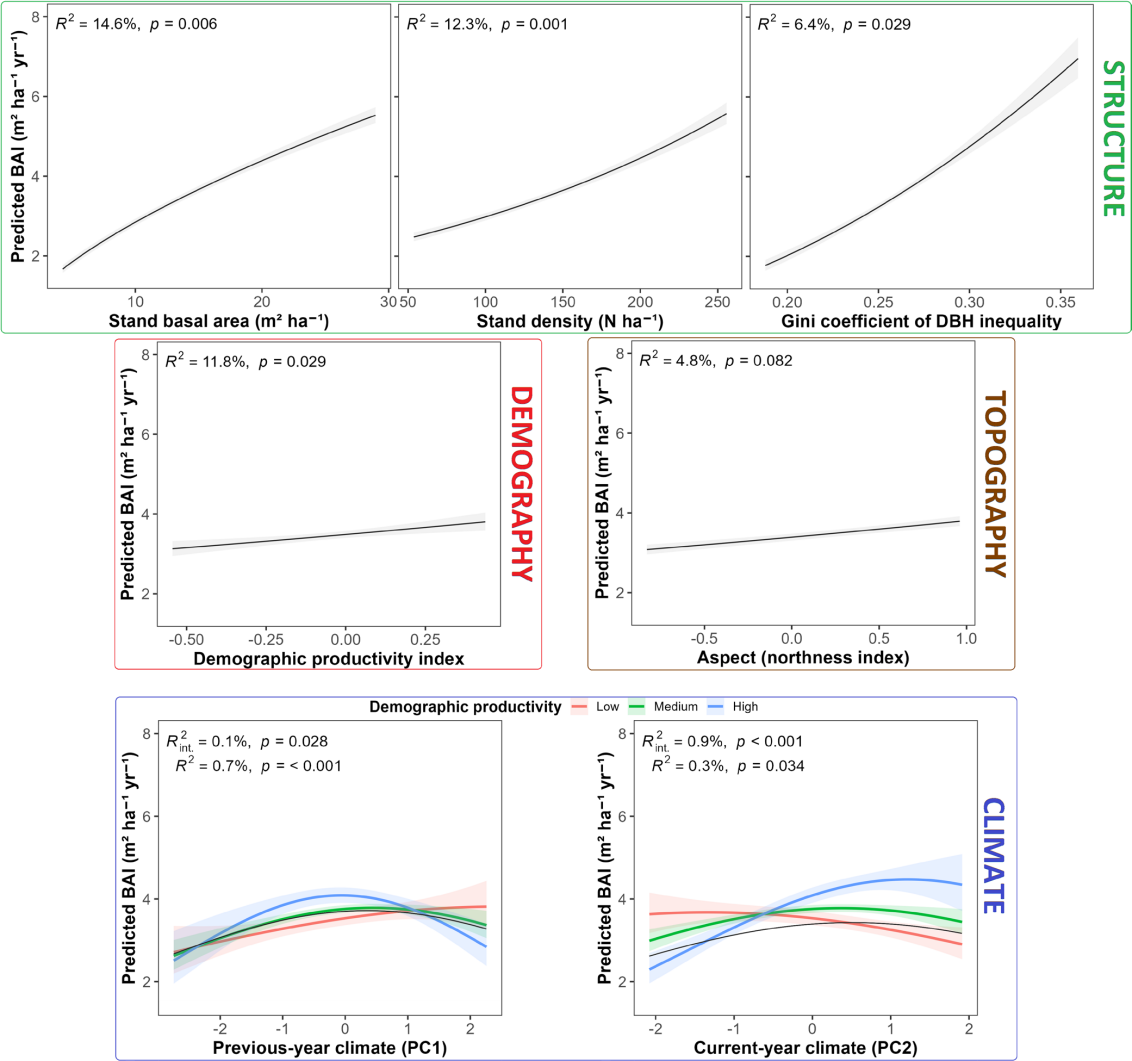
Modelled forest ecosystem productivity drivers under climate warming. Marginal effects of significant standardized predictors and interaction terms on stand productivity (BAI, m^2^ ha^−1^ year^−1^) derived from the generalized mixed‐effects model, shown with 95% confidence intervals (shaded bands). Reported *R* ^2^ and *p*‐values denote semi‐partial coefficients of determination (semi‐partial *R* ^2^) expressed as percentages (%) with their associated probabilities and calculated using the *r2glmm* R package (Jaeger et al. 2017). Colored lines in the first two interaction subplots represent 5th (low), 50th (medium) and 90th (high) quantiles of the demographic productivity index (DPI; with associated *R* ^*2*^_int_ and *p*‐values), illustrating climate‐driven modulation of demographic productivity contribution to forest ecosystem productivity.

Stand structural attributes had the largest effect on forest productivity under recent warming (Figure 7a, Table S2). Stands with higher basal area, greater tree density, and tree size inequality (*β*_2‐4_ = 0.42–0.56; *p <* 0.05) on north‐facing slopes (*β*_6_ = 0.23, *p* ~ 0.08) were significantly more productive. The demographic productivity index also had a strong positive effect on forest productivity (*β*_1_ = 0.55, *p* < 0.05) and high contribution to total model variance (semi‐partial *R* ^2^ = 11.8%). Likelihood‐ratio tests confirmed that including the demographic productivity index substantially improves the explanatory power of the forest ecosystem productivity model (*χ* ^2^ = 70.97, ΔAIC = +58.97, ΔR^2^_m_ = +0.051). Its effect, however, was modulated by climatic variability: deteriorating prior summer conditions reduced the positive effect of demographic productivity (*β*_12_ = −0.03, *p <* 0.05), whereas warmer and wetter current‐year conditions amplified it (*β*_14_ = 0.09, *p <* 0.001), particularly at higher values of the demographic productivity index (90th quantiles). Individual climatic predictors exerted a significant but modest nonlinear effect on stand productivity (semi‐partial *R* ^2^ < 1%; *p <* 0.05): forest productivity increased under moderately warm and moist conditions in both the previous and current year (*β*_8_ = 0.08, *β*_10_ = 0.05), but declined under climatic extremes (*β*_9_ = −0.03, *β*_11_ = −0.06, *p* < 0.05). Collectively, stand structural attributes and demographic performance index accounted for the majority of explained model variance (semi‐partial *R* ^2^ ≈38%), whereas climate and climate–demographic interactions exerted small but statistically significant effects. Age heterogeneity, slope, and biogeographic gradient‐demographic interactions showed no significant effect on forest productivity.

## 4 Discussion

Our results reveal a multifaceted interplay between demographic heterogeneity, forest structure, and regional climatic constraints in shaping European beech growth dynamics under climate warming. Although summer heat stress and moisture deficits emerge as the dominant climatic drivers of beech radial growth, the seasonality and magnitude of growth sensitivity vary across demographic groups and biogeographic regions. Long‐term growth declines are linked to rising summer heat and water stress, and are most pronounced in mid‐sized and large young trees. Multi‐year drought legacies occur only in the driest regions but are largely uniform across the forest strata. At the ecosystem level, demographic divergences lead to contrasting within‐ and between‐regional growth trends under climate warming. Although negative productivity trends are pervasive across the network, regional growth declines are partially buffered by gains in small young (in summer‐mesic regions) and old trees (in summer‐xeric regions). Consequently, forests with higher stand structural complexity and demographic composition dominated by growth‐increasing tree cohorts are more productive under climate warming, but these positive effects are weakened under severe climatic stress. Collectively, these findings demonstrate that demographic heterogeneity plays a crucial role in shaping forest ecosystem responses to climate change and forest productivity trajectories across environmental gradients.

### 4.1 Demographic and Regional Variation in Climate Sensitivity of Beech Radial Growth

Across the study network, beech radial growth is primarily constrained by previous year summer temperature and climatic water balance variation (Figure 2). These findings closely align with published literature on the carry‐over effects of prior adverse growing conditions on radial growth in the following year (e.g., Harvey et al. 2020; Knutzen et al. 2017; van der Maaten et al. 2024). Hot and dry summers exacerbate hydraulic deficits and may cause substantial leaf and bud damage (Neycken et al. 2024; Ognjenović et al. 2022), which inhibits stomatal conductance, reduces photosynthetic activity and, consequently, leads to chronic depletion of carbon reserves for maintaining metabolic homeostasis. Moreover, recurrent summer heatwaves may trigger increased resource allocation towards defensive structures (Leuschner 2020), belowground tissues (e.g., fine roots, Garthen et al. 2025), transient carbohydrate reservoirs (Gérard and Bréda 2014), and/or reproductive organs (Drobyshev et al. 2010)—all of which reduce radial growth the following year. Recent evidence shows an increased frequency of beech masting events across Europe (Hacket‐Pain et al. 2025; Müller‐Haubold et al. 2015). In addition, significant negative growth relationships with spring minimum temperature and mean climatic water balance in xeric regions suggest that early growing season climate variability amplifies growth constraints later in the year if carbon storages are reduced by unfavourable previous year growing conditions. Cambial reactivation and earlywood formation are highly dependent on high carbon reserves (Michelot et al. 2012; Skvareninova et al. 2024), and although warmer and wetter springs may trigger earlier budburst and promote leaf expansion, they concurrently enhance vulnerability to late spring frosts (Rubio‐Cuadrado et al. 2021; Vitasse et al. 2025) and pre‐senescent leaf shedding during summer heat stress, thereby reducing radial growth.

In contrast, beech in more mesic northwestern regions shows stronger and more consistent positive growth relationships with current year spring minimum temperature and mean climatic water balance. Wetter and warmer springs help replenish water and carbon reservoirs in roots and stems early in the growing season, in turn supporting photosynthesis and respiration when summer hydraulic deficits constrain metabolic processes (Hartmann et al. 2020; Ježík et al. 2011; Sever et al. 2025). Beech adopts a conservative stomatal regulation under chronic water stress in order to maintain hydraulic safety and avoid severe xylem dehydration (Arend et al. 2025). This is further supported by the strong positive growth relationships with summer maximum temperature, indicating that the warming‐related phenological shifts that favour earlier cambial reactivation support enhanced wood formation and radial growth in summer months in cold and humid environments. Together, these findings highlight a clear bioclimatic gradient between energy‐driven northwestern (mesic) and moisture‐limited southeastern (xeric) regions, and suggest that radial growth in the former is not yet as limited by climate warming as in the latter, despite evidence of increasing climatic variability and shifts in seasonal climate sensitivity over recent decades (Figure S6b).

Climate sensitivity also differs between demographic groups. Large trees exhibit stronger responses to previous summer temperature variation, whereas small trees show higher sensitivity to spring and summer moisture availability under recent warming (Figure 2b,c). These contrasting responses likely reflect ontogenetic differences in physiological adaptation to shifting climatic constraints (Martínez‐Vilalta et al. 2012; Sanio 1872; Trouillier et al. 2019). Large trees have deeper and more extensive root systems that allow access to broader and deeper soil water levels, but their large size and exposed canopy increase vulnerability to climate stress due to longer hydraulic path lengths and direct irradiance (Bennett et al. 2015; McDowell et al. 2008; Ryan and Yoder 1997; Serra‐Maluquer et al. 2022). Smaller trees, on the other hand, grow buffered from temperature extremes in the subcanopy, but have shallower root systems with resource access limited to the topsoil layer, making them more vulnerable to intraspecific competition and chronic hydraulic stress (Gebauer et al. 2020; Hertel et al. 2013). In addition, positive growth relationships with previous autumn temperature in large trees from mesic regions reveal a high potential for additional late‐season carbon assimilation in canopy‐dominant trees, whereas negative relationships with November CWB in small (mesic regions) and large trees (xeric regions) highlight the role of vertical forest structure and local hydroclimatic conditions on shaping demographic responses to summer moisture deficits (Dox et al. 2020; Tumajer et al. 2025).

Despite these functional differences between large and small trees, we do not observe significantly different growth responses to extreme summer droughts between demographic groups, but rather region‐dependent drought legacies (Figure 3). Beech in mesic regions did not exhibit significant growth deviations during or following severe droughts, although its rising sensitivity to prior and current maximum summer temperatures suggests a rising sensitivity to summer heat stress over recent decades (Figure S6b). In xeric regions, both large and small trees show comparable multi‐year growth legacies, reflecting how extreme summer droughts synchronize growth responses across the forest strata, even though small young trees exhibited relatively weaker post‐drought growth deviations. Large trees have more stringent stomatal controls but larger carbon reserves than small trees, allowing them to compensate for elevated respiration costs and reduced photosynthesis under chronic hydraulic stress at the expense of radial growth (González de Andrés et al. 2021; Leuschner 2020; Zhang et al. 2025). When large trees reduce water and nutrient uptake under drought stress, neighboring small trees may benefit from reduced resource competition in the upper soil layer, which could explain the comparatively weaker RWI deviations of small young trees (Pretzsch et al. 2018; Rubio‐Cuadrado et al. 2025).

These results collectively demonstrate that climatic sensitivity of beech radial growth varies across the demographic strata and biogeographic gradients. Although ontogenetic differences modulate tree growth sensitivity to climate variability, the magnitude and persistence of drought impacts on tree growth are contingent on local hydraulic constraints. Rising growth sensitivity to deteriorating summer climatic conditions across the network suggest that beech acclimation to future climatic perturbations will increasingly depend on the frequency, severity, and duration of climatic extremes (Leifsson et al. 2024), and post‐drought recovery of small trees (Bréda et al. 2006; Yu et al. 2022), particularly at the warm and dry edge of the species' distribution.

### 4.2 Demographic Divergences in Regional Growth Trends Under Climate Warming

Long‐term regional growth trajectories strongly diverged across the demographic strata and biogeographic regions since the onset of accelerated climate warming (i.e., 1980s; Figure 4). Recent growth declines were most pronounced in mid‐sized and large trees from the Dinaric Mts. (i.e., CRO, BOS and ALB), which is consistent with previous findings from dry‐marginal beech populations (e.g., Dulamsuren et al. 2017; Hacket‐Pain et al. 2016; Sánchez‐Gómez and Aranda 2024; Serrano‐Notivoli et al. 2025) and predictive growth models (e.g., Bošela et al. 2023; Klesse et al. 2024; Martinez del Castillo et al. 2022). These regions are extremely xeric despite high annual precipitation due to the typical karst geology (i.e., shallow and nutrient poor soils with low water‐holding capacity atop of highly porous bedrock), which exacerbates soil moisture deficits and tree vulnerability to hydraulic failure (Eilmann et al. 2014; Rukh et al. 2023). Large trees are particularly vulnerable to chronic hydraulic stress due to enhanced size‐related constraints on hydraulic conductance (Phillips et al. 2016; Ryan and Yoder 1997; Trouillier et al. 2019). In addition, CRO is amongst the oldest populations in the study network (Table 1), so age‐related senescence in photosynthetic capacity may be an additional contributor to the observed growth declines (Aranda et al. 2015; Zang et al. 2014). The fact that regional declines began around the 1960s across the demographic strata, however, suggests a long‐term deterioration of tree hydraulic function across the region (Aertsen et al. 2014; Arend et al. 2022), and may indicate decreasing ecosystem resilience to climate change (Cailleret et al. 2019; Gillner et al. 2013).

In contrast to the drought‐driven growth declines in the Dinaric Mts., several northwestern regions exhibited positive growth trends under climate warming, particularly among small and mid‐sized young trees, and higher average productivity compared to the pre‐warming period (Figure 4; Figure S7a). Beech is a highly shade‐tolerant species capable of persisting under prolonged canopy suppression until conditions become favourable for a rapid vertical release (Bolte et al. 2014; Di Filippo et al. 2012; Pavlin et al. 2024). In mesic environments, small trees benefit from warming‐driven alleviation of cold limitation and extension of the growing season (Roibu et al. 2022; Schurman et al. 2024), as summer water availability is not yet a major growth constraint. This interpretation is also consistent with the stronger positive growth associations with current year climatic variation identified in the climate response analysis (Figure 2a, Figure S6). Earlier phenological onset and shifting competitive interactions within the forest strata likely concurrently contribute to the observed growth trends. Nevertheless, considerable within‐ and between‐regional variation in growth dynamics indicates that even in mesic environments, not all trees benefit from positive warming effects (Figure S7b,c).

Not all regional growth patterns followed the biogeographic gradient. We also observed significant negative growth trends at the core distribution range (i.e., CZ), and positive growth trends in more xeric southeastern regions (i.e., BUL and S ROM), despite these latter regions being one of the driest in the network (Table 1.; MAP__annual_ < 1000 mm, CWB__SUMMER_ < 65 mm/yr)., Local soil conditions (i.e., low micronutrient availability) and historical legacies (i.e., high air pollution in the 1990s; Oulehle et al. 2024) have likely amplified the negative effects of recent climatic pressures on tree growth in the Jizera Mts (CZ). Moreover, these populations are structurally dominated by large young trees (Figure 6a; over 75% trees > 50 cm and < 200 years), which are less resilient to rising environmental stress.

On the other hand, beech populations in the Balkan Mts. (BUL) and in the southern Carpathians (S ROM) likely acquired higher resistance to drought‐induced hydraulic stress through long‐term adaptive genetic differentiation and acclimation through structural adjustments in anatomical and morphological traits (e.g., larger leaf area, broader fine root systems; Adamič et al. 2023, 2025; Arnič et al. 2021; Gričar et al. 2024; Prislan et al. 2018), which concurrently increase phenotypic plasticity under chronically dry conditions. Similar observations were previously noted at the dry margin of species' distribution in southern (e.g., Muffler et al. 2020; Tegel et al. 2014) and Central Europe (Archambeau et al. 2020; Cavin and Jump 2017; Schuldt et al. 2016). The prevalence of very old trees (> 300 years) in these regions and their recent growth increase affirms their successful acclimation to warmer and drier conditions. Contrary to the expected senescence‐related growth decline, these trees still show ample room for increased carbon uptake, and exhibit functional traits associated with conservative water use and enhanced carbon storage (Liu et al. 2017; Puchi et al. 2024) that may promote long‐term resilience to climatic stress (Meyer et al. 2021; Tao et al. 2024). By contrast, beech in relatively more mesic northern Carpathians (N ROM) exhibited significant growth declines despite more favourable hydroclimatic conditions, likely reflecting a limited expression of adaptive hydraulic and biomechanical functional traits that consequently constrain beech productivity under rising climatic stress.

Overall, long‐term growth trends were strongly associated with rising summer temperatures and declining climatic water balance along ecological gradients, particularly in large old and small mid‐aged trees (Figure 5c). These patterns confirm that severe heat and drought exposure during months of peak cambial activity (i.e., June–July) are universal drivers of beech growth declines across the species distribution range (Etzold et al. 2022; Unterholzner et al. 2024; van der Maaten et al. 2024). The fact that majority of demographic groups across the network exhibited lower average productivity under climate warming compared to the pre‐warming period (Figure S7a) suggests that recent positive growth trends in small and mid‐sized young and old trees may be short‐term under intensifying climatic pressures. Moreover, the fact that the strongest absolute growth trends were observed in large young trees highlights their disproportional vulnerability under intensifying heat and drought stress. The large within‐ and between‐regional heterogeneity in growth dynamics underscores the importance of accounting for demographic composition and local climatic conditions when inferring about future forest ecosystem trajectories under climate change.

### 4.3 Demographic Heterogeneity and Structural Complexity Modulate Forest Productivity Under Climate Warming

Demographic variability in growth dynamics is an important contributor to forest productivity trends under climate warming. Regional productivity “losers” (i.e., regions with negative net productivity) generally exhibit consistent negative growth trends across the demographic strata, whereas regional “winners” (i.e., regions with positive net productivity) show strong compensatory productivity contributions of small young and mid‐sized young and old trees, respectively (Figure 6a). This demographic buffering effect is even more evident across the biogeographic gradient (Figure 6b), which carries broad consequences for forest productivity, carbon storage, and ecosystem dynamics. Rapid ascension of previously suppressed trees may partially offset carbon losses associated with the declines of large trees, by sustaining biomass accumulation within the forest strata, in turn facilitating continuous forest ecosystem productivity in regions where climatic conditions remain within the species optimal physiological thresholds. In some xeric regions (i.e., S ROM and BUL), positive net productivity trends are also supported by gains in large mid‐aged and small old trees. Large old trees are key features of primary beech forests that serve as vital long‐term carbon reservoirs and drivers of functional and structural diversity, and may act as important demographic mediators of forest growth dynamics under shifting climatic conditions (Dúhová et al. 2025; Kozák et al. 2023; Lutz et al. 2018).

Model results show that stand structural complexity and demographic variability in growth dynamics, rather than the individual impacts of climatic forcing, shape forest ecosystem productivity under climate warming (Figure 7, Table S2). Forest stands with higher basal area, denser canopies, and greater tree size heterogeneity are substantially more productive, reflecting how structural complexity improves resource partitioning and microclimatic buffering capacity in natural beech forests (Bončina et al. 2025; Ruiz‐Benito et al. 2014; Weigel et al. 2023). In addition, forest stands with a larger proportion of demographic cohorts with positive growth trajectories relative to their structural representation exhibit higher productivity. However, the positive effects of demographic heterogeneity are modulated by interannual climate variability and markedly decline under extreme climatic stress, indicating that compensatory structural mechanisms are contingent on local climatic stochasticity, and may only partially offset beech forest productivity declines under chronic climatic stress. This buffering capacity may be further constrained in highly fragmented forest ecosystems that recently experienced high‐severity disturbances, but this inference remains outside the scope of this study.

The fact that small trees in northwestern regions and large trees in southeastern regions exhibit productivity gains under climate warming carries important implications for future beech ecosystem dynamics. Key uncertainties regarding future forest trajectories are associated with the putative expectations that the predicted rise in tree mortality will decrease longevity and accelerate carbon loss beyond gains, which would ultimately constrain long‐term sequestration and reduce forest ecosystem carbon storage capacity (Brienen et al. 2020; Büntgen et al. 2019; Seidl et al. 2017; Tumajer et al. 2025). Recurrent droughts and heatwaves have already intensified hydraulic and thermal constraints on tree physiological processes, and if extremes events occur more frequently and become more persistent, widespread growth declines and mortality might follow (McDowell et al. 2020; Munné‐Bosch 2018; Wolf and Paul‐Limoges 2023). However, recent evidence from temperate and subalpine forest ecosystems suggest that post‐disturbance legacy structures and warming‐driven phenological shifts may sustain long‐term carbon sink function by enhancing forest structural complexity, advancing understory growth and increasing carbon storage in living biomass (Gough et al. 2019; Marqués et al. 2023; Wang et al. 2023; Zohner et al. 2023). Whether these compensatory growth dynamics represent a transient response to intensifying climatic disturbances (e.g., Forzieri et al. 2021; Patacca et al. 2023; Senf et al. 2018) or reflect long‐term ecosystem stability (e.g., Carnicer et al. 2021; Cerioni et al. 2025; Janda et al. 2025) remains uncertain.

Nonetheless, our empirical findings align with mechanistic predictions from vegetation model ensembles showing that demographic variability in growth dynamics modulates forest ecosystem responses to climate change (Eckes‐Shephard et al. 2025; Fisher et al. 2018). Integrating demographic variability and structural heterogeneity into large‐scale predictive growth models may reduce uncertainties associated with predictions of future ecosystem trajectories and carbon dynamics, particularly in unmanaged forests where demographic structure plays a crucial role for ecosystem functioning and stability. Our findings suggest that future beech productivity will increasingly depend on the interaction between forest structure, demographic variability in growth dynamics, and local environmental conditions across biogeographic gradients. Long‐term monitoring of primary beech forests will therefore be essential for understanding how demographic heterogeneity mediates the impacts of intensifying climatic stress on forest ecosystem productivity across ecological gradients.

## 5 Conclusion

Systematic assessments of spatial and demographic variability in growth dynamics are essential for understanding how structural heterogeneity shapes forest ecosystem responses to climate change and, subsequently, for improving forecasts of future ecosystem trajectories under intensifying climate‐driven disturbances. Yet intraspecific empirical studies of demographic variability across broad ecological gradients are currently missing.

Our findings show that European beech does not respond uniformly to recent climatic variation across biogeographic regions and demographic strata. Instead, growth responses to recent warming vary substantially across demographic groups and environmental gradients, with recent growth declines largely driven by mid‐sized and large trees, and strongly associated with warmer and drier summer conditions. Pronounced drought legacies occur mainly in the driest regions, where hydroclimatic constraints on radial growth are most severe. These demographic divergences in climate sensitivity translate into contrasting within‐ and between‐regional productivity trends under climate warming. Although warming‐driven ecosystem productivity declines are partially offset by productivity gains in small and mid‐aged and old tree cohorts in mesic and xeric regions, respectively, this compensatory effect is largely contingent on local climatic stochasticity and limited in offsetting widespread productivity losses across landscapes.

Forest structural complexity and demographic heterogeneity enhance ecosystem productivity under favourable climatic conditions, but have a limited buffering capacity under severe climatic stress. Future beech ecosystem productivity trajectories will increasingly depend on the interaction between physiological tree growth legacies, demographic structural variability, and local microsite conditions across ecological gradients. Predictive growth models should explicitly account for demographic and structural heterogeneity as key modulators of beech growth dynamics to improve projections of future ecosystem productivity and carbon dynamics under climate change.

## Author Contributions

**Jakob Pavlin:** conceptualization, data curation, writing – review and editing. **Krešimir Begović:** conceptualization, data curation, investigation, formal analysis, visualization, writing – original draft, methodology. **Thomas Langbehn:** data curation, writing – review and editing. **Jakub Kašpar:** writing – review and editing. **Kristyna Svobodová Langbehn:** data curation, writing – review and editing. **Thomas A. Nagel:** data curation, writing – review and editing. **Jeňýk Hofmeister:** writing – review and editing. **Miloš Rydval:** writing – review and editing. **Pavel Janda:** writing – review and editing, data curation. **Andrei Popa:** writing – review and editing. **Martin Mikoláš:** writing – review and editing, data curation. **Daniel Kozak:** writing – review and editing, data curation. **Miroslav Svoboda:** data curation, supervision, funding acquisition, writing – review and editing, project administration. **Stjepan Mikac:** writing – review and editing, data curation.

## Funding

Funding for this research was provided by the Czech Science Foundation Grant (project no. 15‐14840S, 21‐27454S, 24‐12210K, and 25‐18519S). Additionally, JK was supported by the Czech Science Foundation Grant (GAČR 21‐47163L), whereas AP was supported by the FOR‐CLIMSOC Programme (project no. PN23090204 and PN23090302), financed by the Ministerul Cercetării, Inovării și Digitalizării in Romania.

## Ethics Statement

The study was performed in accordance with the ethical standards of the Institutional and International Research committee and with the 1964 Helsinki Declaration and its later amendments or comparable ethical standards.

## Conflicts of Interest

The authors declare no affiliations with or involvement in any organization or entity that could have a financial interest in the subject matters discussed within this manuscript.

## Supporting information


**Table S1:** Summary of regional climate‐growth correlations.
**Table S2:** Summary of the forest ecosystem productivity model.
**Figure S1:** Long‐term regional growth trajectories of non‐overlapping age classes and age.
**Figure S2:** Long‐term regional growth trajectories of non‐overlapping size classes and size.
**Figure S3:** Long‐term climatic conditions across the study network.
**Figure S4:** Comparison of observed and modelled basal area increment stand chronologies.
**Figure S5:** Monthly and seasonal climate‐growth relationships over three distinct time periods.
**Figure S6:** Spatiotemporal variation in regional climate‐growth relationships.
**Figure S7:** Regional and demographic variability in growth dynamics under climate warming.
**Figure S8:** Forest ecosystem productivity model diagnostics.

## Data Availability

All datasets supporting the findings of this study are publicly available in Dryad (Begović et al. 2026) at https://doi.org/10.5061/dryad.8kprr4z20.
