# Supplementary material for: Demographic and Structural Variability Modulate Growth Dynamics in European Beech Primary Forests
Source: Glob Chang Biol. 2026 May 6;32:e70902. doi: 10.1111/gcb.70902 (PMC13147310; doi:10.1111/gcb.70902)
Supplement: Supplementary file 1 — Table S1: Summary of regional climate‐growth correlations. Table S2: Summary of the forest ecosystem productivity model. Figure S1: Long‐term regional growth trajectories of non‐overlapping age classes and age. Figure S2: Long‐term regional growth trajectories of non‐overlapping size classes and size. Figure S3: Long‐term climatic conditions across the study network. Figure S4: Comparison of observed and modelled basal area increment stand chronologies. Figure S5: Monthly and seasonal climate‐growth relationships over three distinct time periods. Figure S6: Spatiotemporal variation in regional climate‐growth relationships. Figure S7: Regional and demographic variability in growth dynamics under climate warming. Figure S8: Forest ecosystem productivity model diagnostics. [file GCB-32-e70902-s001.pdf]

SUPPORTING INFORMATION

Demographic and structural variability modulate growth dynamics in European beech primary forests

Krešimir Begović[1], Jakob Pavlin[1], Thomas Langbehn[1], Kristyna Svobodová Langbehn[1,2], Jakub Kašpar[3], Andrei Popa[4,5], Thomas A. Nagel[1,6], Jeňýk Hofmeister[1], Pavel Janda[1], Miloš Rydval[1], Daniel Kozak[1], Martin Mikoláš[1], Stjepan Mikac[7], Miroslav Svoboda[1]

1 Faculty of Forestry and Wood Sciences, Czech University of Life Sciences Prague, Czechia

2 Faculty of Environmental Sciences, Czech University of Life Sciences Prague, Czechia

3 Department of Forest Ecology, Landscape Research Institute, Lidická 25/27, 602 00 Brno, Czech Republic

4 National Institute for Research and Development in Forestry "Marin Drăcea", Bucharest, Romania

5 Faculty of Silviculture and Forest Engineering, Transilvania University of Brasov, Brasov, Romania

6 Biotechnical faculty, University of Ljubljana

7 Department of Forest Ecology and Silviculture, Faculty of Forestry, University of Zagreb, Croatia

S1. Study caveats

Despite the general robustness of our multi-factorial approach, several methodological considerations should be acknowledged. Growth trend and climate response analysis are seldom plagued by inherent biases related to field data collection strategies and the general nature of radial growth time-series (Nehrbass-Ahles et al., 2014; Visser et al., 2023). However, our data collection adopted a population-wide random sampling design, obtaining information from both living and dead trees across a broad range of tree ages and sizes, in turn ensuring a representative selection for cross-regional stratified comparisons. In addition, using a linear mixed-effects model to account for potential age- and size-effects (incl. log-transformation of tree size and BAI *a priori*) allowed us to minimize the inherent multicollinearity between tree age and size, and explore the spatial patterns across different demographic cohorts (Klesse & Bigler, 2025).

Although differences in stratification schemes between growth trend and climate response analysis make comparability between short-term climate sensitivity and long-term growth trends difficult (due to differences in total number of trees represented), regression analysis bridged the potential gap between the two datasets and confirmed there were no discrepancies that would confound results and interpretation of either analysis. Additional uncertainties regarding climate response and superposed epoch analysis are associated with the nature of extracted climate data and misrepresentation of local climatic conditions, evapotranspiration estimates and the selection of drought years. To test whether this was a significant issue, climate response analysis and SEA were repeated using climate information from the KNMI Climate Explorer obtained as CRU TS 4.08 gridded 0.5° instrumental climate dataset (https://climexp.knmi.nl/start.cgi; Harris et al., 2020). Results revealed no significant relative differences between the two climate datasets. Although local drought timing and intensity varied among forest stands and may not be entirely

adequately captured by climatic values extrapolated from gridded datasets, our approach to identifying drought years based on standardized climatic anomalies rather than RW reductions provides a more objective basis for cross-regional comparisons in epochal anomalies.

Finally, our forest productivity mixed-effects model did not explicitly account for the effects of other fine-scale edaphic factors, such as bedrock, soil type and soil moisture, disturbance legacies, or competitive interactions, all of which may exacerbate climate influences on tree- and stand-level productivity (*e.g.*, Buras, 2017). However, our research aims were designed to disentangle broad stand- and regional-level productivity patterns, rather than fine-scale variation at the plot- and tree-level, where the aforementioned drivers exert a stronger effect. The potential confounding effects of sample size variations across stands and demographic cohorts were addressed by relatively downweighing the impacts of under-represented groups or stands on final model predictions.

References

Buras, A., 2017. A comment on the expressed population signal. Dendrochronologia 44, 130–132. https://doi.org/10.1016/j.dendro.2017.03.005

Harris, I., Osborn, T.J., Jones, P., Lister, D., 2020. Version 4 of the CRU TS monthly high-resolution gridded multivariate climate dataset. Sci Data 7, 109. https://doi.org/10.1038/s41597-020-0453-3

Klesse, S., Bigler, C., 2025. Growth trends in basal area increments: The underlying problem, consequences for research and best practices. Dendrochronologia 90, 126296. https://doi.org/10.1016/j.dendro.2025.126296

Nehrbass-Ahles, C., Babst, F., Klesse, S., Nötzli, M., Bouriaud, O., Neukom, R., Dobbertin, M., Frank, D., 2014. The influence of sampling design on tree-ring-based quantification of forest growth. Glob Change Biol 20, 2867–2885. https://doi.org/10.1111/gcb.12599

Visser, H., van der Maaten-Theunissen, M., Maaten, E. van der, 2023. BAI BAI bias – An evaluation of uncertainties in calculating basal area increments from cores. Dendrochronologia 78, 126066. https://doi.org/10.1016/j.dendro.2023.126066

Tables

Table S1. Summary of regional climate-growth correlations.

Values represent the mean ± standard deviation (max) of Pearson correlation coefficients (*r*) between residual RWI chronologies and climate variables (CWB, PREC, Tmax, Tmin,) across the network. Results are grouped by climate parameter, month, and time period (1940–1979, 1980–2020, and 1940–2020). **Asterisks** indicate months with the strongest and spatially broadest (***), broad (**) and significant (*) correlations after false discovery rate (FDR) adjustment. Capital letters denote months of the current growing year, otherwise are shown months of the year prior to tree ring formation.

Metric	Month (previous/CURRENT YEAR)	1940-1979	1980-2020	1940-2020
CWB	Jun	0.013 ± 0.143 (0.22)	**0.004 ± 0.262 (0.43)***	**0 ± 0.172 (-0.27)***
CWB	Jul	**0.187 ± 0.122 (0.31)***	**0.246 ± 0.103 (0.36)*****	**0.22 ± 0.083 (0.3)*****
CWB	Aug	**0.086 ± 0.158 (0.4)***	**0.134 ± 0.178 (0.26)***	**0.137 ± 0.159 (0.32)***
CWB	Sep	0.028 ± 0.146 (0.34)	0.018 ± 0.099 (-0.16)	0.013 ± 0.074 (0.12)
CWB	Oct	0.084 ± 0.094 (0.29)	0.008 ± 0.172 (-0.25)	0.035 ± 0.095 (0.17)
CWB	Nov	**0.059 ± 0.175 (0.25)***	**-0.223 ± 0.084 (-0.37)****	-0.09 ± 0.09 (-0.18)
CWB	Dec	0.019 ± 0.112 (-0.17)	-0.012 ± 0.154 (-0.28)	-0.021 ± 0.052 (0.1)
CWB	JAN	-0.013 ± 0.093 (-0.15)	-0.008 ± 0.12 (0.31)	-0.013 ± 0.065 (-0.11)
CWB	FEB	**-0.147 ± 0.059 (-0.21)***	-0.018 ± 0.106 (0.21)	**-0.1 ± 0.056 (-0.13)***
CWB	MAR	-0.041 ± 0.115 (0.2)	**-0.104 ± 0.157 (-0.34)***	-0.079 ± 0.114 (0.19)
CWB	APR	**0.163 ± 0.115 (0.35)***	**0.09 ± 0.171 (0.25)***	**0.113 ± 0.089 (0.23)***
CWB	MAY	**0.087 ± 0.164 (0.22)***	-0.022 ± 0.056 (-0.12)	0.02 ± 0.089 (-0.14)
CWB	JUN	0.002 ± 0.154 (0.36)	-0.072 ± 0.083 (-0.22)	-0.022 ± 0.088 (-0.14)
CWB	JUL	-0.073 ± 0.112 (-0.35)	-0.028 ± 0.205 (-0.5)	-0.036 ± 0.101 (-0.24)
CWB	AUG	0.013 ± 0.128 (-0.23)	-0.098 ± 0.117 (-0.18)	**-0.071 ± 0.09 (-0.19)***
CWB	SEP	-0.119 ± 0.134 (-0.28)	-0.047 ± 0.145 (-0.24)	-0.108 ± 0.097 (-0.18)
CWB	OCT	**-0.115 ± 0.14 (-0.29)***	0.016 ± 0.131 (0.26)	-0.023 ± 0.075 (0.14)
CWB	p_summer	**0.206 ± 0.214 (0.39)****	**0.14 ± 0.207 (0.5)***	**0.153 ± 0.192 (0.41)****
CWB	WINTER	-0.091 ± 0.086 (-0.19)	-0.017 ± 0.127 (0.31)	-0.077 ± 0.052 (-0.11)
CWB	SPRING	**0.148 ± 0.135 (0.29)***	**-0.036 ± 0.196 (0.34)****	**0.032 ± 0.159 (0.31)***
CWB	SUMMER	0.005 ± 0.145 (-0.29)	-0.068 ± 0.125 (-0.38)	-0.058 ± 0.102 (-0.2)
CWB	GS	-0.03 ± 0.13 (-0.21)	-0.069 ± 0.084 (-0.2)	-0.041 ± 0.081 (-0.2)
PREC	Jun	-0.007 ± 0.16 (-0.26)	**0 ± 0.244 (0.4)***	**-0.012 ± 0.178 (-0.28)***
PREC	Jul	**0.191 ± 0.123 (0.31)****	**0.265 ± 0.106 (0.37)*****	**0.22 ± 0.089 (0.32)*****
PREC	Aug	0.049 ± 0.15 (0.37)	**0.128 ± 0.189 (0.3)***	**0.113 ± 0.154 (0.33)***
PREC	Sep	0.046 ± 0.146 (0.33)	0.037 ± 0.093 (-0.18)	0.031 ± 0.074 (0.14)
PREC	Oct	0.09 ± 0.091 (0.25)	-0.008 ± 0.164 (-0.25)	0.048 ± 0.087 (-0.13)
PREC	Nov	**0.044 ± 0.176 (0.25)***	**-0.214 ± 0.078 (-0.37)****	-0.085 ± 0.079 (-0.18)

PREC	Dec	0.019 ± 0.118 (-0.17)	-0.037 ± 0.144 (0.24)	-0.004 ± 0.049 (0.1)
PREC	JAN	0.009 ± 0.089 (0.18)	-0.006 ± 0.121 (0.31)	-0.004 ± 0.065 (-0.1)
PREC	FEB	**-0.157 ± 0.089 (-0.25)****	-0.021 ± 0.121 (0.22)	-0.103 ± 0.07 (-0.18)
PREC	MAR	-0.062 ± 0.116 (0.2)	-0.111 ± 0.151 (-0.34)	-0.097 ± 0.118 (-0.2)
PREC	APR	**0.181 ± 0.13 (0.4)***	**0.095 ± 0.161 (0.26)***	**0.134 ± 0.072 (0.23)***
PREC	MAY	**0.104 ± 0.158 (0.23)***	-0.027 ± 0.049 (-0.09)	0.055 ± 0.084 (-0.14)
PREC	JUN	0.006 ± 0.158 (0.37)	-0.075 ± 0.077 (-0.18)	-0.014 ± 0.092 (-0.15)
PREC	JUL	-0.079 ± 0.113 (-0.34)	-0.018 ± 0.196 (-0.46)	-0.035 ± 0.104 (-0.23)
PREC	AUG	0.002 ± 0.124 (-0.24)	-0.118 ± 0.134 (-0.24)	**-0.066 ± 0.1 (-0.23)***
PREC	SEP	-0.134 ± 0.122 (-0.28)	-0.034 ± 0.135 (-0.29)	-0.1 ± 0.086 (-0.18)
PREC	OCT	**-0.126 ± 0.137 (-0.29)***	0.014 ± 0.122 (0.23)	**-0.041 ± 0.083 (-0.16)***
PREC	p_summer	**0.18 ± 0.207 (0.37)***	**0.165 ± 0.2 (0.5)***	**0.131 ± 0.186 (0.42)****
PREC	WINTER	-0.095 ± 0.089 (-0.19)	-0.027 ± 0.141 (0.32)	-0.065 ± 0.067 (-0.15)
PREC	SPRING	**0.165 ± 0.13 (0.28)***	-0.038 ± 0.182 (0.34)	0.055 ± 0.149 (0.3)
PREC	SUMMER	0.002 ± 0.144 (-0.28)	-0.078 ± 0.125 (-0.33)	-0.066 ± 0.103 (-0.21)
PREC	GS	-0.041 ± 0.124 (-0.2)	-0.068 ± 0.084 (-0.21)	-0.041 ± 0.083 (-0.19)
Tmax	Jun	**-0.131 ± 0.157 (0.27)***	**-0.036 ± 0.202 (-0.34)***	**-0.103 ± 0.148 (0.21)***
Tmax	Jul	**-0.239 ± 0.158 (-0.37)***	**-0.275 ± 0.174 (-0.63)*****	**-0.197 ± 0.116 (-0.47)****
Tmax	Aug	**-0.197 ± 0.192 (-0.58)****	-0.087 ± 0.122 (-0.33)	**-0.133 ± 0.103 (-0.32)***
Tmax	Sep	**-0.108 ± 0.189 (-0.27)***	-0.098 ± 0.136 (-0.27)	**-0.031 ± 0.116 (-0.19)***
Tmax	Oct	**0.345 ± 0.122 (0.49)*****	**-0.012 ± 0.209 (-0.3)***	**0.199 ± 0.12 (0.32)****
Tmax	Nov	0.125 ± 0.12 (0.28)	0.036 ± 0.143 (0.28)	0.087 ± 0.114 (0.23)
Tmax	Dec	**0.03 ± 0.146 (0.3)***	0.094 ± 0.133 (0.36)	0.086 ± 0.065 (0.18)
Tmax	JAN	0.066 ± 0.164 (0.39)	**0.118 ± 0.148 (0.33)***	**0.142 ± 0.053 (0.2)***
Tmax	FEB	0.038 ± 0.125 (-0.22)	-0.049 ± 0.101 (-0.21)	0.014 ± 0.1 (-0.22)
Tmax	MAR	**0.096 ± 0.159 (0.36)***	**0.04 ± 0.241 (0.4)***	**0.026 ± 0.178 (0.32)***
Tmax	APR	**-0.14 ± 0.091 (-0.28)***	-0.086 ± 0.144 (-0.35)	**-0.102 ± 0.078 (-0.31)***
Tmax	MAY	0.01 ± 0.096 (0.16)	0.021 ± 0.155 (-0.37)	0.007 ± 0.115 (-0.25)
Tmax	JUN	-0.025 ± 0.167 (-0.34)	0.003 ± 0.134 (-0.2)	-0.056 ± 0.063 (-0.15)
Tmax	JUL	0.149 ± 0.113 (0.26)	**0.125 ± 0.256 (0.61)***	**0.137 ± 0.172 (0.4)***
Tmax	AUG	0.023 ± 0.083 (0.12)	**0.053 ± 0.147 (0.29)***	0.048 ± 0.073 (0.21)
Tmax	SEP	0.032 ± 0.163 (0.33)	**0.113 ± 0.183 (0.49)***	**0.101 ± 0.156 (0.39)***
Tmax	OCT	**-0.132 ± 0.153 (-0.21)***	-0.031 ± 0.083 (-0.16)	-0.043 ± 0.066 (-0.15)
Tmax	p_summer	**-0.26 ± 0.214 (-0.39)***	**-0.204 ± 0.169 (-0.49)***	**-0.181 ± 0.155 (-0.42)***
Tmax	WINTER	0.048 ± 0.088 (0.23)	**0.109 ± 0.145 (0.22)***	0.098 ± 0.06 (0.15)
Tmax	SPRING	-0.015 ± 0.129 (-0.22)	0.002 ± 0.159 (-0.25)	-0.027 ± 0.108 (-0.23)
Tmax	SUMMER	**0.054 ± 0.16 (-0.22)***	0.025 ± 0.156 (0.42)	**0.068 ± 0.14 (0.32)***
Tmax	GS	-0.062 ± 0.084 (-0.17)	**0.074 ± 0.13 (0.27)***	0.034 ± 0.07 (0.13)
Tmin	Jun	-0.087 ± 0.16 (-0.26)	-0.087 ± 0.084 (-0.19)	-0.064 ± 0.098 (-0.23)
Tmin	Jul	**-0.14 ± 0.115 (-0.31)***	**-0.2 ± 0.213 (-0.49)****	**-0.167 ± 0.13 (-0.32)***
Tmin	Aug	**-0.175 ± 0.156 (-0.55)***	**-0.028 ± 0.197 (-0.34)***	**-0.119 ± 0.098 (-0.24)***

Tmin	Sep	**0.019 ± 0.188 (0.34)***	-0.023 ± 0.128 (-0.22)	**0.049 ± 0.142 (-0.21)***
Tmin	Oct	**0.419 ± 0.115 (0.49)*****	**0.095 ± 0.265 (0.39)****	**0.228 ± 0.103 (0.35)****
Tmin	Nov	0.176 ± 0.132 (0.35)	-0.007 ± 0.157 (-0.24)	**0.066 ± 0.128 (0.23)***
Tmin	Dec	**0.14 ± 0.158 (0.39)***	0.094 ± 0.131 (0.27)	**0.096 ± 0.049 (0.19)***
Tmin	JAN	0.057 ± 0.158 (0.34)	**0.146 ± 0.177 (0.31)***	**0.113 ± 0.051 (0.17)***
Tmin	FEB	-0.041 ± 0.117 (-0.26)	-0.059 ± 0.102 (-0.24)	-0.038 ± 0.102 (-0.25)
Tmin	MAR	**0.19 ± 0.202 (0.51)***	**-0.022 ± 0.274 (0.44)***	**0.115 ± 0.224 (0.41)****
Tmin	APR	-0.066 ± 0.14 (-0.24)	0.078 ± 0.179 (-0.29)	-0.034 ± 0.097 (-0.2)
Tmin	MAY	0.037 ± 0.112 (0.23)	-0.008 ± 0.158 (-0.34)	0.031 ± 0.095 (-0.14)
Tmin	JUN	-0.069 ± 0.168 (-0.4)	**-0.073 ± 0.166 (-0.29)***	-0.087 ± 0.058 (-0.16)
Tmin	JUL	0.124 ± 0.141 (0.24)	**0.136 ± 0.266 (0.49)***	**0.113 ± 0.2 (0.37)***
Tmin	AUG	0.066 ± 0.121 (0.29)	**0.026 ± 0.18 (0.31)***	**0.034 ± 0.131 (0.31)***
Tmin	SEP	-0.003 ± 0.16 (-0.41)	**0.058 ± 0.197 (0.37)***	**0.056 ± 0.162 (-0.32)***
Tmin	OCT	**-0.189 ± 0.127 (-0.36)***	-0.12 ± 0.084 (-0.22)	**-0.134 ± 0.078 (-0.25)***
Tmin	p_summer	**-0.129 ± 0.179 (-0.35)***	**-0.118 ± 0.194 (-0.41)***	-0.107 ± 0.148 (-0.37)
Tmin	WINTER	0.053 ± 0.091 (0.21)	**0.11 ± 0.166 (-0.24)***	0.08 ± 0.065 (0.13)
Tmin	SPRING	**0.174 ± 0.218 (0.44)***	**0.009 ± 0.244 (-0.44)***	**0.056 ± 0.19 (0.31)***
Tmin	SUMMER	**0.073 ± 0.211 (0.35)***	**0.031 ± 0.174 (0.32)***	**0.016 ± 0.167 (0.35)***
Tmin	GS	-0.072 ± 0.141 (-0.29)	**0.109 ± 0.206 (-0.39)***	-0.013 ± 0.135 (-0.28)

Table S2. Summary of the forest ecosystem productivity model.

$$\log(BAI+0.001) = \beta_0 + \beta_1 \cdot DPI + \beta_2 \cdot \text{Stand basal area} + \beta_3 \cdot \text{Stand density} + \beta_4 \cdot \text{Gini DBH} + \beta_5 \cdot \text{Gini age} + \beta_6 \cdot \text{Aspect} + \beta_7 \cdot \text{Slope} + \beta_8 \cdot \text{PC1 linear} + \beta_9 \cdot \text{PC1 quadratic} + \beta_{10} \cdot \text{PC2 linear} + \beta_{11} \cdot \text{PC2 quadratic} + \beta_{12} \cdot (DPI \times \text{PC1 linear}) + \beta_{13} \cdot (DPI \times \text{PC1 quadratic}) + \beta_{14} \cdot (DPI \times \text{PC2 linear}) + \beta_{15} \cdot (DPI \times \text{PC2 quadratic}) + \beta_{16} \cdot (DPI \times \text{Biogeographic gradient (xeric vs mesic) I}) + u_{region} + u_{stand:region} + u_{year} + \varepsilon$$

Predictor	Est. (SE)	z	*p*	semi-partial R^2 (95% CI)
**(Intercept)**	-	**7.332**	**< .001*** **	-
**Demographic productivity index (DPI)**	**.551 (.252)**	**2.184**	**< .029***	**11.8 (8.5-15.5)**
**Stand density (N/ha)**	**.411 (.125)**	**3.287**	**< .001*** **	**12.3 (9.0-16.0)**
**Stand basal area (m^2/ha)**	**.547 (.198)**	**2.755**	**.006*** **	**14.6 (11.0-18.4)**
**DBH inequality (Gini)**	**.559 (.256)**	**2.186**	**.029***	**6.4 (3.9-9.4)**
Age inequality (Gini)	.105 (.234)	0.451	.652	0.3 (0.0-1.3)
**Aspect (northness index)**	**.231 (.133)**	**1.737**	**.082***	**4.8 (2.7-7.6)**
Slope (°)	-.269 (.175)	-1.537	.124	4.4 (2.3-7.0)
**Previous-year climate (linear, PC1)**	**.084 (.019)**	**4.514**	**< .001*** **	**0.7 (0.1-2.1)**
**Previous-year climate (quadratic, PC1)**	**-.034 (.016)**	**-2.072**	**< .038***	**0.5 (0.0-1.7)**
**Current-year climate (linear, PC2)**	**.054 (.026)**	**2.116**	**< .034*** **	**0.3 (0.0-1.3)**
**Current-year climate (quadratic, PC2)**	**-.068 (.018)**	**-3.707**	**< .001*** **	**0.5 (0.0-1.7)**
**DPI × Previous-year climate (lin.)**	**-.029 (.013)**	**-2.199**	**.028***	**0.1 (0.0-1.8)**
DPI × Previous-year climate (quad.)	.012 (.014)	0.862	.388	0.0 (0.0-0.5)
**DPI × Current-year climate (lin.)**	**.091 (.013)**	**7.182**	**< .001*** **	**0.9 (0.1-2.3)**
DPI × Current-year climate (quad.)	.004 (.013)	0.292	.771	0.0 (0.0-0.5)
DPI × Biogeographic gradient	-.309 (.218)	-1.416	.157	3.1 (1.4-5.3)
**Random effects**	**τ**	**χ2**	***p***	
Stand (Region)	.141	3.063	**.080***	-
Region	.129	319.72	**< .001*** **	-
Year	-	268.262	**< .001*** **	-

Whole model	AIC	χ2	RMSE	*p*	R^2_m / R^2_c
	383.051	128.43	.253	**< .001*** **	.446 / .903

The table shows standardized regression coefficients (Est.) of fixed predictors with their associated standard errors (SE), Wald z-values (z), probabilities (p) from the generalized linear mixed-effects model fitted with a Gaussian error structure. Semi-partial coefficients of determination (semi-partial R^2; %) quantify the unique contribution of individual predictors to the model explanatory power calculated using the r2glmm R package (Jaeger et al., 2017). The response variable represents mean stand-level basal area increment (m^2 ha^{-1} yr^{-1}), log-transformed to reduce right skewness and to accommodate zero values. The intercept (β_0) and slope parameters (β_1–β_{16}) correspond to model-estimated fixed effects. .Random effects show the hierarchical data structure with random intercepts (u) for stands nested within regions and repeated annual observations, while ϵ represents the residual error. Random effects variance components (τ) are shown for each random effect. Likelihood ratio tests statistics (χ^2) and associated probabilities are given for random effect parameters and the whole model. Average prediction error (RMSE), marginal (R^2_m) and conditional determination coefficients (R^2_c) are also tabulated. Asterisks indicate significance levels: *p < .1, **p < .01, and ***p < .001, respectively.

Figures

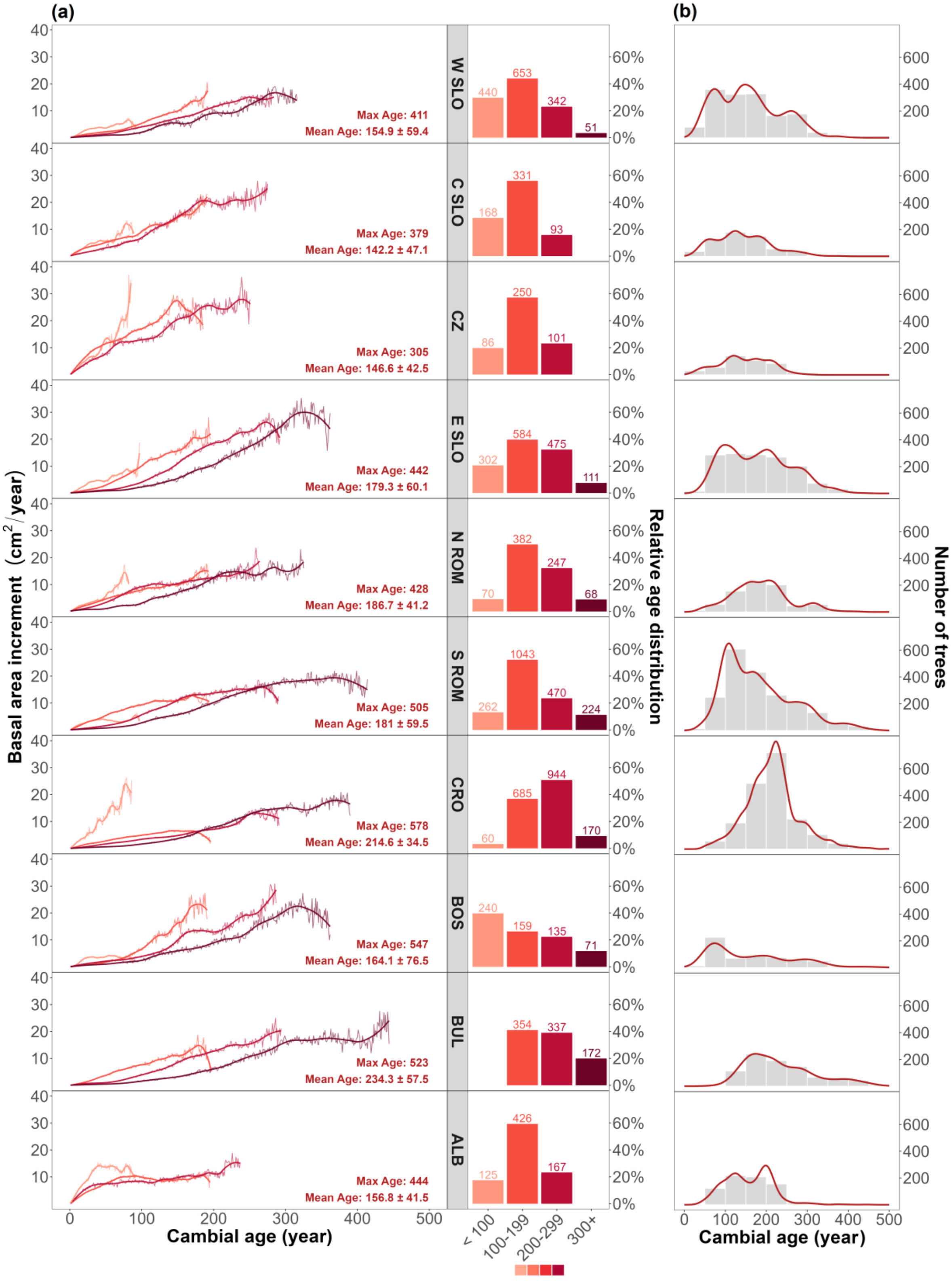

(a)
(b)
Basal area increment (cm²/year)
Cambial age (year)
Relative age distribution
Number of trees
W SLO
Max Age: 411
Mean Age: 154.9 ± 59.4
440
653
342
51
C SLO
Max Age: 379
Mean Age: 142.2 ± 47.1
168
331
93
CZ
Max Age: 305
Mean Age: 146.6 ± 42.5
86
250
101
E SLO
Max Age: 442
Mean Age: 179.3 ± 60.1
302
584
475
111
N ROM
Max Age: 428
Mean Age: 186.7 ± 41.2
70
382
247
68
S ROM
Max Age: 505
Mean Age: 181 ± 59.5
262
1043
470
224
CRO
Max Age: 578
Mean Age: 214.6 ± 34.5
60
685
944
170
BOS
Max Age: 547
Mean Age: 164.1 ± 76.5
240
159
135
71
BUL
Max Age: 523
Mean Age: 234.3 ± 57.5
354
337
172
ALB
Max Age: 444
Mean Age: 156.8 ± 41.5
125
426
167
< 100
100-199
200-299
300+

Fig. S1. Long-term regional growth trajectories of non-overlapping age classes and age structure.

(**a**) Annual basal area increment (BAI) regional chronologies of non-overlapping 100-yr age classes with bar plots showing the relative age distribution across regions. Smoothed curves are plotted using generalized additive models with cubic regression splines (*gam*) for easier visual interpretation. Regional chronologies are truncated to the minimum number of trees based on Cohen's D power analysis over the 1940-2020 period. Numbers atop each bar denote the absolute number of trees per age class. (**b**) Regional age distribution showing absolute tree counts as 50-yr bins overlaid with the kernel density estimate (**red line**). Regional chronologies and bar plots are arranged along the summer moisture (CWB) gradient. Regional acronyms denote regions as defined in Table 1.

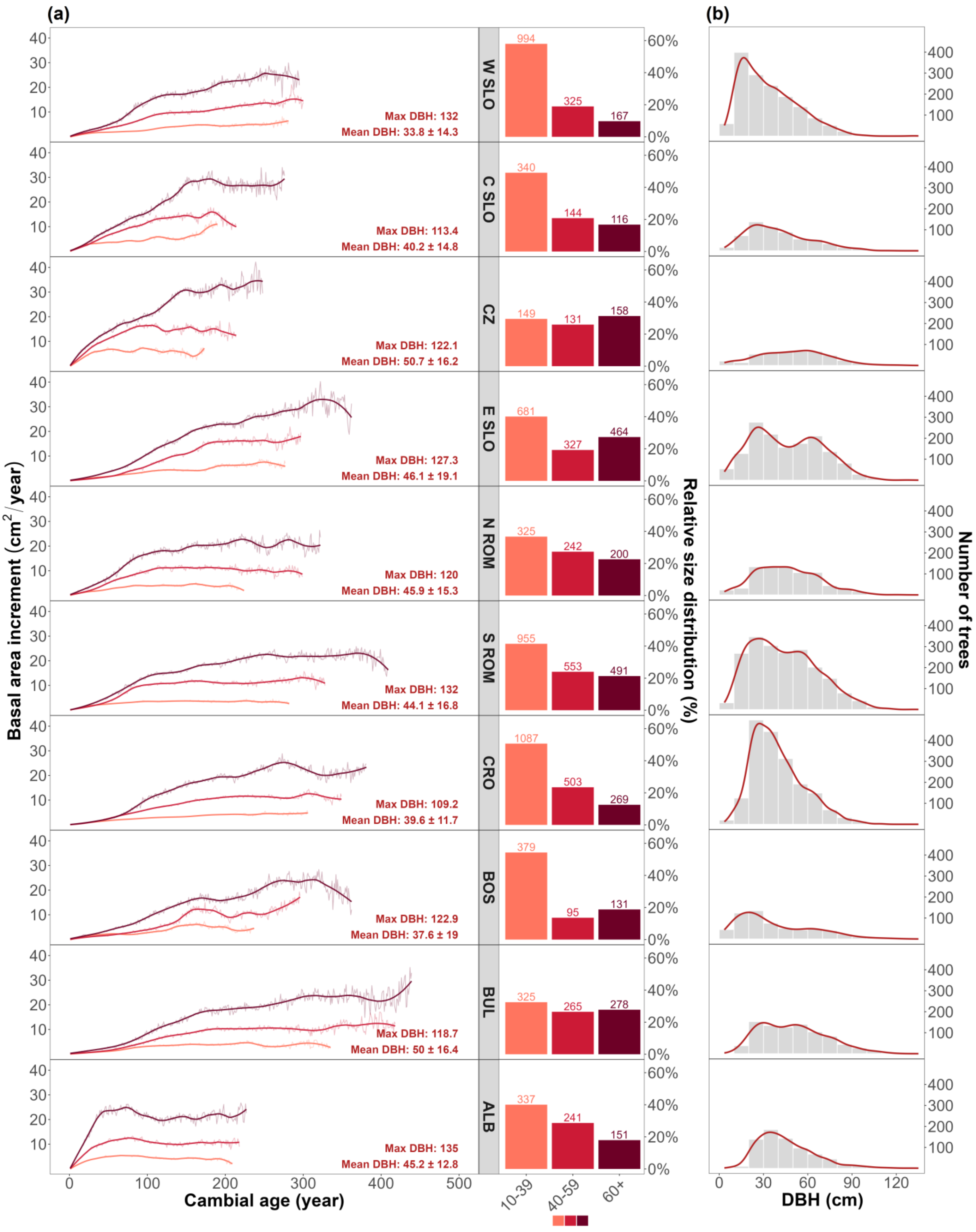

(a)
(b)
Basal area increment (cm²/year)
Cambial age (year)
Relative size distribution (%)
Number of trees
DBH (cm)
W SLO
Max DBH: 132
Mean DBH: 33.8 ± 14.3
994
325
167
C SLO
Max DBH: 113.4
Mean DBH: 40.2 ± 14.8
340
144
116
CZ
Max DBH: 122.1
Mean DBH: 50.7 ± 16.2
149
131
158
E SLO
Max DBH: 127.3
Mean DBH: 46.1 ± 19.1
681
327
464
N ROM
Max DBH: 120
Mean DBH: 45.9 ± 15.3
325
242
200
S ROM
Max DBH: 132
Mean DBH: 44.1 ± 16.8
955
553
491
CRO
Max DBH: 109.2
Mean DBH: 39.6 ± 11.7
1087
503
269
BOS
Max DBH: 122.9
Mean DBH: 37.6 ± 19
379
95
131
BUL
Max DBH: 118.7
Mean DBH: 50 ± 16.4
325
265
278
ALB
Max DBH: 135
Mean DBH: 45.2 ± 12.8
337
241
151
10-39
40-59
60+

Fig. S2. Long-term regional growth trajectories of non-overlapping size classes and size structure.

(**a**) Annual basal area increment (BAI) regional chronologies of non-overlapping 10-cm size classes with bar plots showing the relative size distribution across regions. Smoothed curves are plotted using generalized additive models with cubic regression splines (*gam*) for easier visual interpretation. Regional chronologies are truncated to the minimum number of trees based on Cohen's D power analysis over the 1940-2020 period. Numbers atop each bar denote the absolute number of trees per size class. (**b**) Regional size distribution showing absolute tree counts as 10-cm bins overlaid with the kernel density estimate (**red line**). Regional chronologies and bar plots are arranged along the summer moisture (CWB) gradient. Regional acronyms denote regions as defined in Table 1.

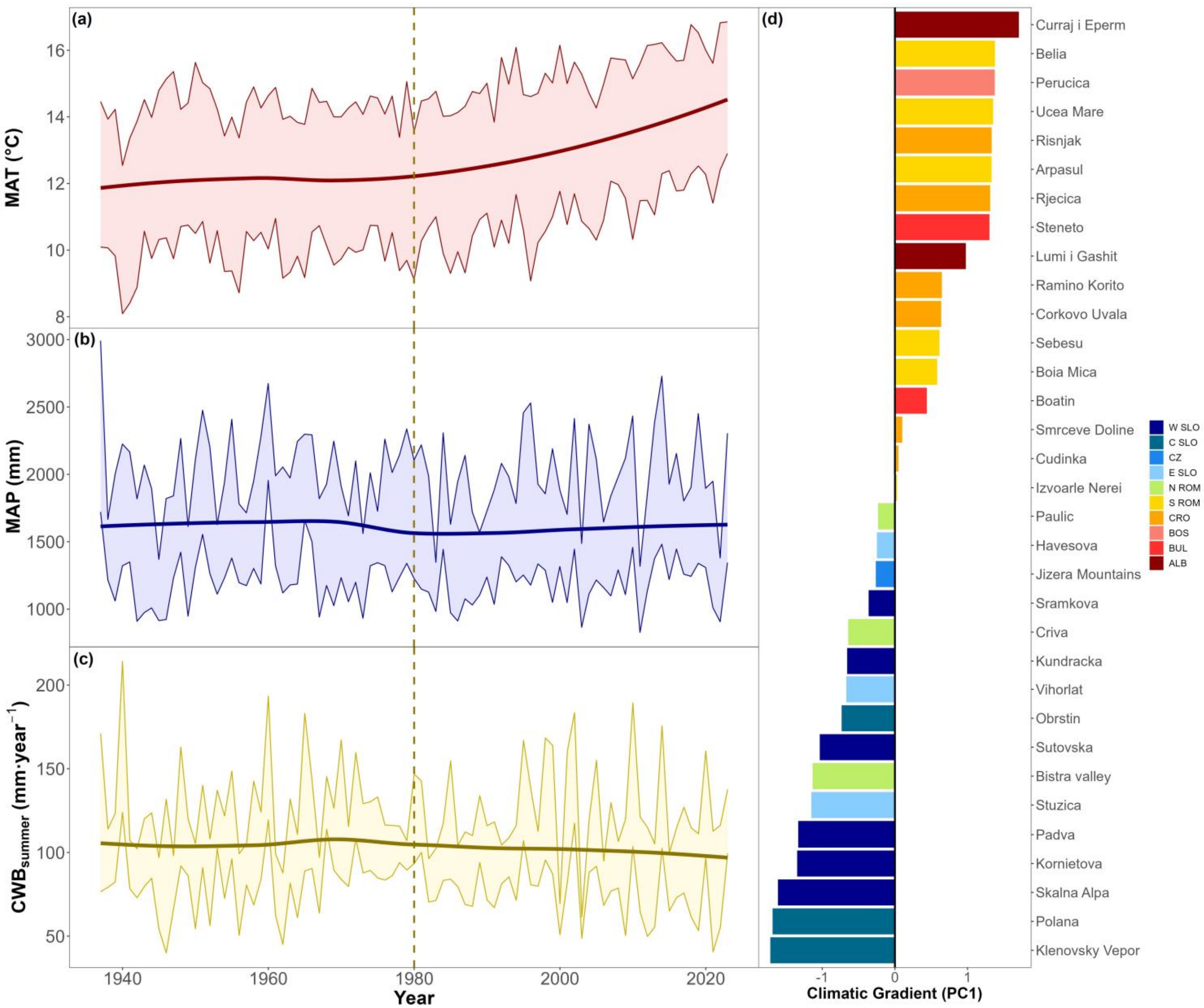

Fig. S3. Long-term climatic conditions across the study network.

(**a**) Mean annual temperature (MAT), (**b**) precipitation totals (MAP) and (**c**) summer climatic water balance (CWB) of individual forest stands over the 1940-2020 period. Smoothed curves are plotted using generalized additive models with cubic regression splines (*gam*) for easier visual interpretation and represent averages of each climatic factor. Vertical dashed line denotes the onset of accelerated global warming. (**d**) Climatic gradient (Principal Component; PC1) of forest stands and regions based on average summer climatic conditions since the onset of accelerated climate warming (*i.e.*, 1980–2020). Regional acronyms denote regions as defined in Table 1.

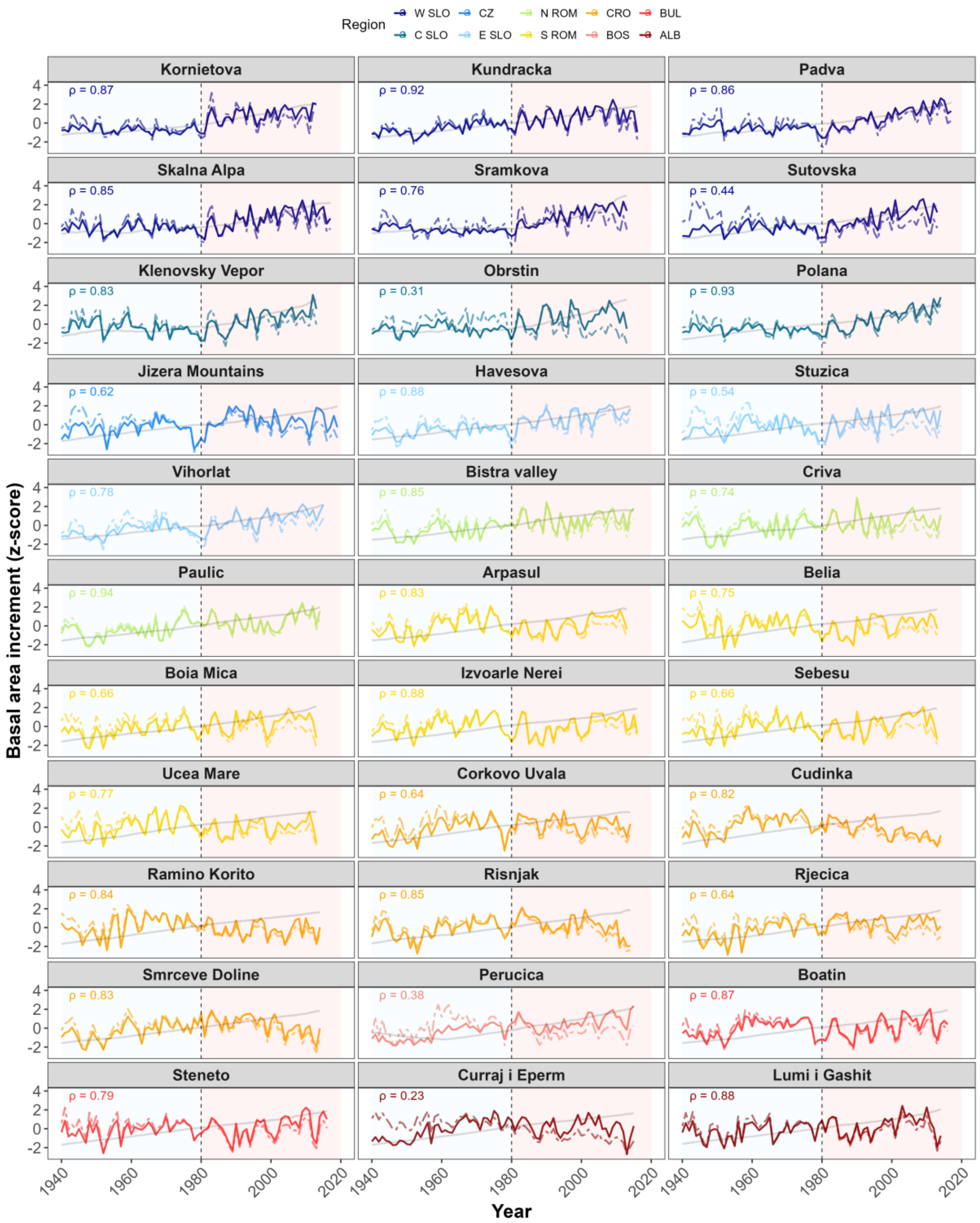

Fig. S4. Comparison of observed and modelled basal area increment stand chronologies.

Observed (**solid line**) and predicted (**dashed**) stand-level BAI anomalies (z-score, i.e., mean = 0, SD = 1) since the mid-20th century. The thin grey line denotes the size-expected BAI trend. Spearman's ρ denotes the monotonic agreement between the observed and predicted BAI anomalies. Chronologies are arranged latitudinally (southwards) along the summer moisture gradient (CWB_{summer}). Regional acronyms denote regions as defined in Table 1.

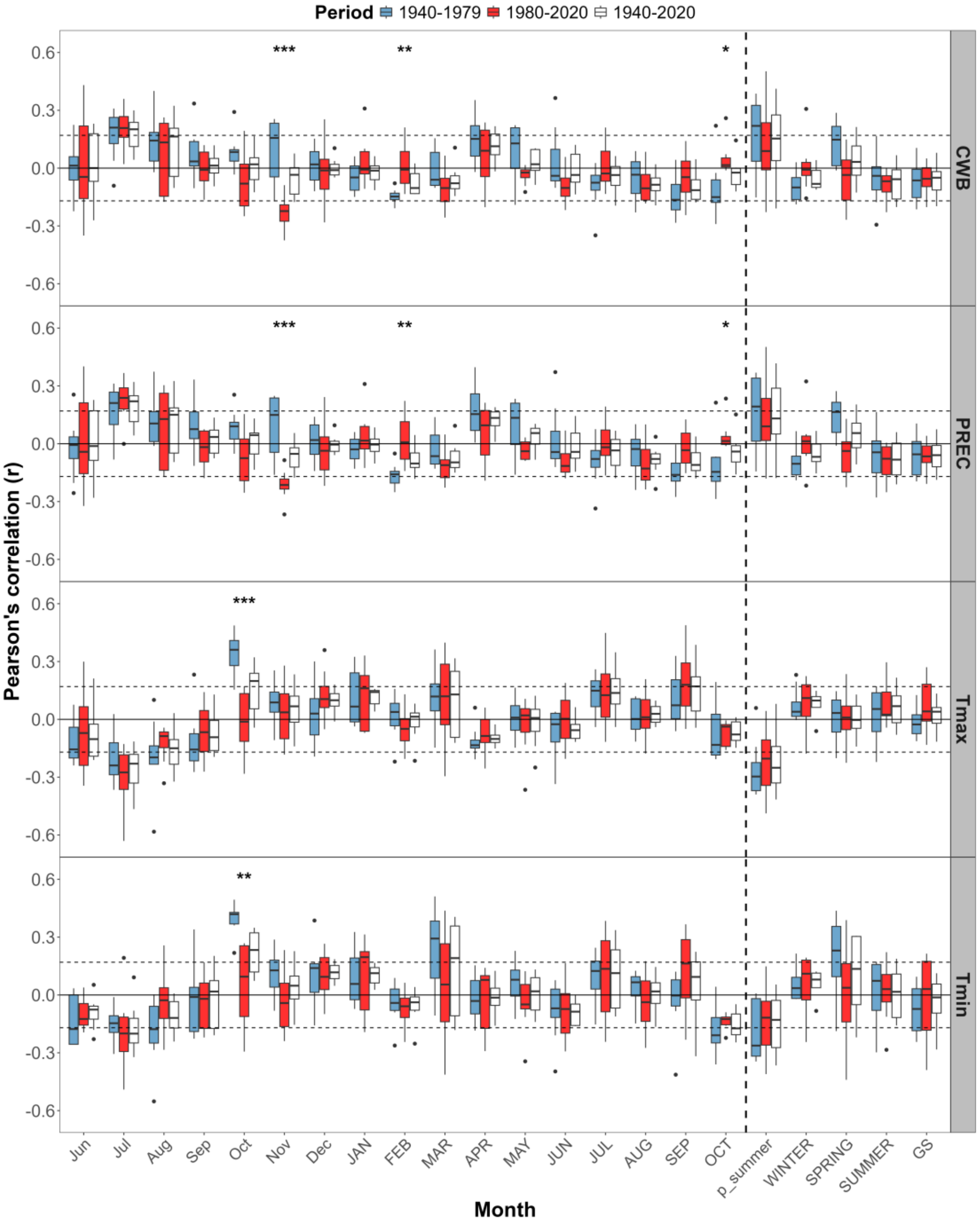

Fig. S5. Monthly and seasonal climate-growth relationships over three distinct time periods.

Whisker boxplots denote Pearson's correlations between stand-level RWI chronologies and monthly/seasonal climatic factors (CWB = climatic water balance, PREC = precipitation totals, and Tmin/max = minimum and maximum temperature). Boxplots show the interquartile range and the median correlation coefficients. Whiskers span from minimum to maximum values. The asterisks denote months with significant changes in *r* values between time periods. Vertical dashed line separates the individual months from the seasonal aggregates. Capital letters denote months of the current growing year, otherwise are shown months of the year prior to tree ring formation.

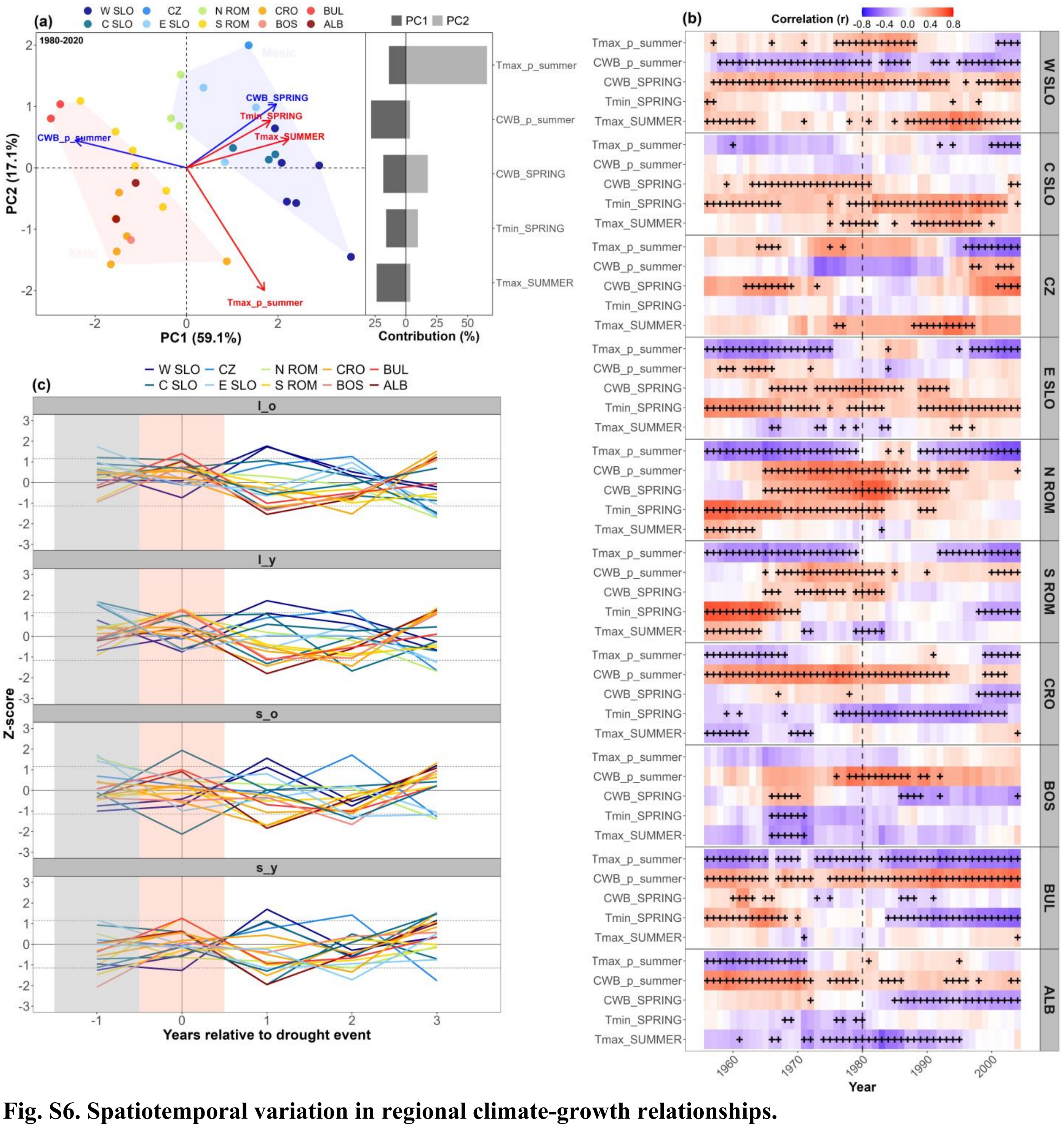

Fig. S6. Spatiotemporal variation in regional climate-growth relationships.

(**a**) PCA biplots of regional patterns in seasonal climate-growth relationships after the onset of accelerated climate warming (1980-2020). Contributions of the strongest seasonal climatic factors to the first two PCs are denoted. (**b**) 21-year running correlations between regional RWI chronologies and the strongest growth-limiting climate factors (Fig. S3): maximum summer temperature of

the current (*Tmax_SUMMER*) and previous (*Tmax_p_summer*) year, minimum spring temperature (*Tmin_SPRING*), and mean previous summer and current spring climatic water balance (*pCWB_summer, CWB_SPRING*). Significance at $p < 0.05$ is indicated by plus signs. **(c)** Superposed epochal deviations of scaled (z-scored) residual stand-level chronologies of demographic groups during extreme drought years (1946, 1950, 1956, 1961, 1992, 2000, 2007). Horizontal dashed lines indicate 95th percentile bootstrapped significance thresholds.

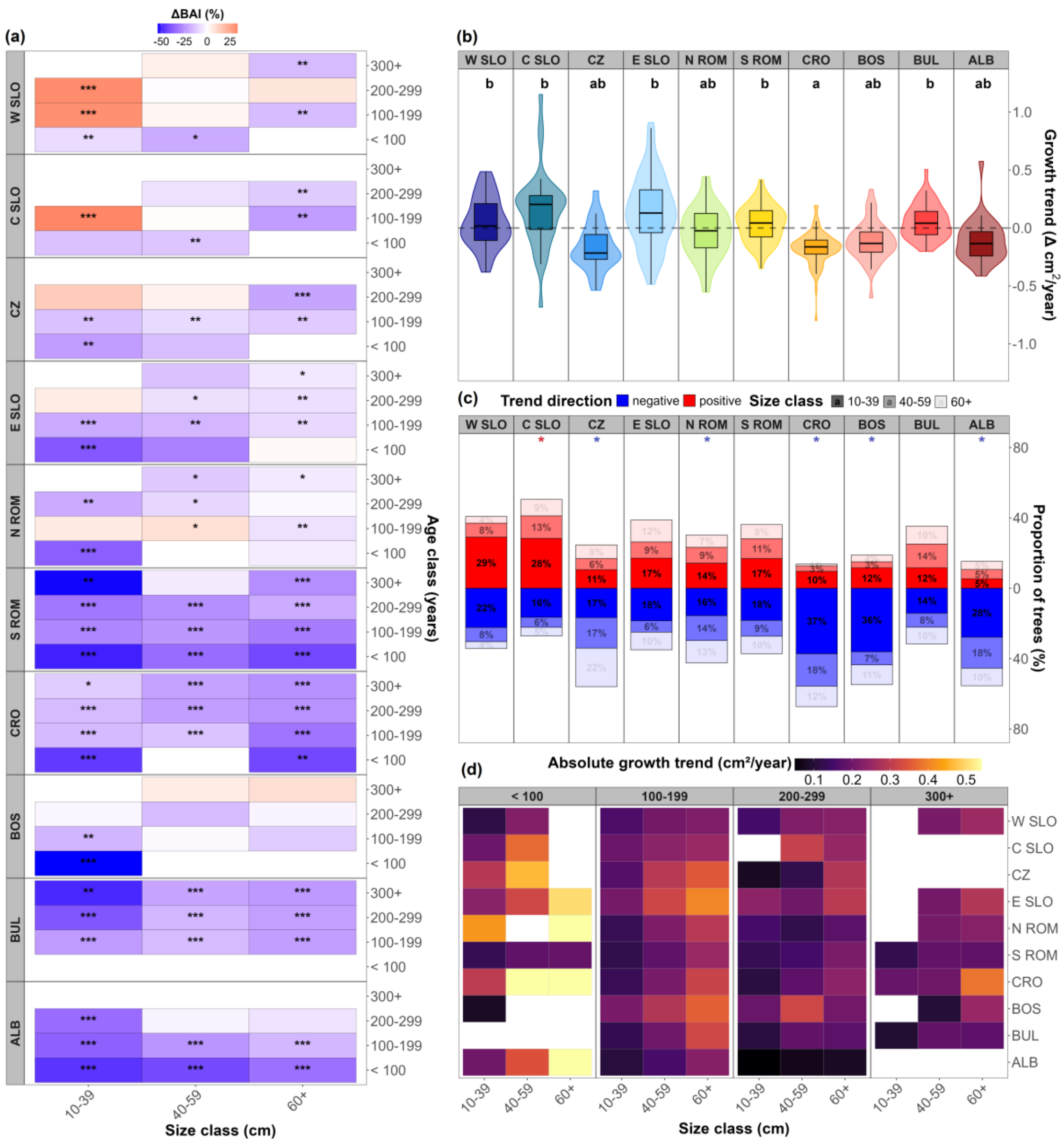

Fig. S7. Regional and demographic variability in growth dynamics under climate warming.

(**a**) Heatmap of the relative change (%) in median log-response ratios (*lnRR*) of BAI_{std} across demographic groups between two time periods (*i.e.*, 1980-2020 vs 1940-1979). Red and blue tiles denote higher/lower BAI_{std} in the more recent period. Asterisks denote statistical significance based on the Wilcoxon signed-rank tests ($p < 0.05$). (**b**) Violin plots of median Sen's slope values (cm^2/year) per region. Box-plots denote median values and standard interquartile ranges, whereas letters denote statistical grouping based on ANOVA with BH-adjusted post-hoc test. (**c**) Mirrored bar plots of tree distribution with significant positive (**red**) and negative (**blue**) growth trends across regions and size classes. Asterisks indicate significant within-regional differences between positive and negative tree proportions based on the Chi-squared test. (**d**) Heatmap of tree-count–weighted mean absolute Sen's slope (cm^2/year) for demographic groups and region. Regional acronyms denote regions as defined in Table 1.

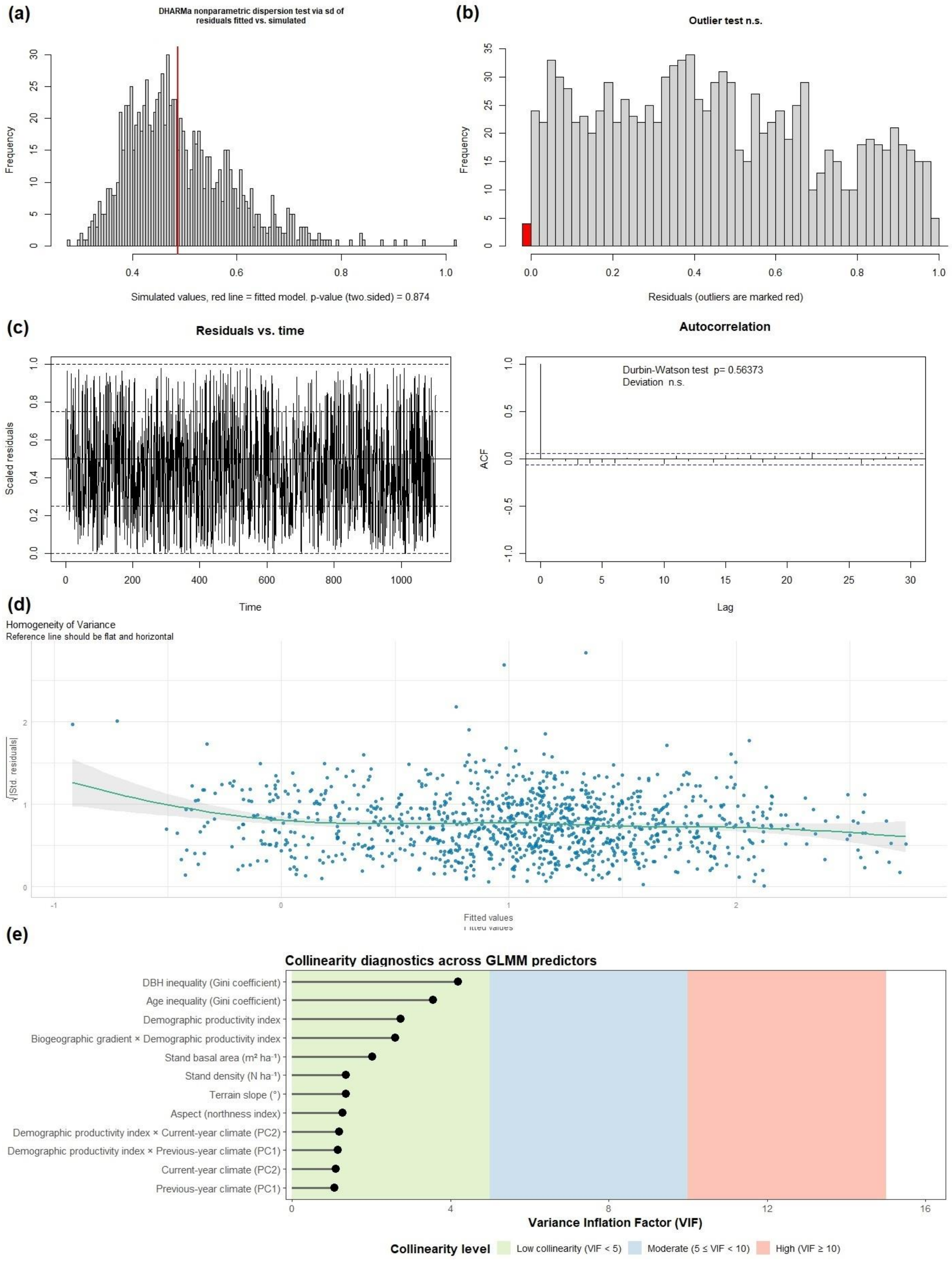

Fig. S8. Forest ecosystem productivity model diagnostics.

Shown are plots of key GLMM model diagnostic elements, incl. normality (**a**), outlier test (**b**), autocorrelation (**c**), homoscedasticity (**d**), and multi-collinearity (**e**).
